# Effect of Huang-Lian Jie-Du Decoction on Glucose and Lipid Metabolism in Type 2 Diabetes Mellitus: A Systematic Review and Meta-Analysis

**DOI:** 10.3389/fphar.2021.648861

**Published:** 2021-04-29

**Authors:** Zhipeng Hu, Maoyi Yang, Ya Liu, Qiyue Yang, Hongyan Xie, Sihan Peng, Juan Gao, Chunguang Xie

**Affiliations:** Hospital of Chengdu University of Traditional Chinese Medicine, Chengdu, China

**Keywords:** meta-analysis, systematic review, type diabetes mellitus, traditional Chinese medicine, huang-lian jie-du decoction

## Abstract

**Background:** Type 2 diabetes mellitus (T2DM) is a heterogeneous disease characterized by persistent hyperglycemia. Huang-Lian Jie-Du decoction (HLJDD) is a traditional Chinese medicine formula which is widely used in treating T2DM in China. A thorough understanding of current body of evidence is needed.

**Objective:** this study aims to summarize the clinical evidence of HLJDD for T2DM to provide an up-to-date and accurate understanding of this issue for research and clinical practice.

**Methods:** Six databases were searched from inception to June 27, 2020 without language and publication status restrictions and randomized controlled trials about HLJDD on T2DM were included. Two evaluators searched and screened citations independently. Risk of bias was assessed by 2019 version 2 of the Cochrane risk-of-bias tool for randomized trials (RoB2). Risk ratio (RR) with 95% confidence interval (CI) was used as an effect measure for dichotomous outcomes and mean difference (MD) with 95% CI was used for continuous outcomes. Subgroup analyses and sensitivity analyses were carried out.

**Results:** Nine studies including 811 participants were included in this study. The overall risk of bias was high risk. Compared with metformin alone, combination treatment of HLJDD and metformin may result in a reduction in HbA1c, FBG, 2hPG, HOMA-IR and an improved lipid metabolism. Evidence comparing HLJDD and metformin or no intervention or placebo was insufficient. The quality of evidence was low.

**Conclusions:** Current evidence about HLJDD on T2DM is still uncertain and more high-quality studies are needed to firmly establish the clinical efficacy and safety of HLJJD.

## Introduction

Type 2 diabetes mellitus (T2DM) is a heterogeneous disease characterized by persistent hyperglycemia as main symptom and insulin resistance as pathophysiological feature. In recent years, although some studies have shown a gradual slowdown of incidence, the prevalence of T2DM continues to rise (Magliano, et al., 2019). Currently, there are more than 425 million diabetic patients in the world, 90% of which are T2DM (Cho et al., 2018). China has the largest diabetic population in the world (Li et al., 2020). Chronic complications caused by T2DM have a significant impact on the health and quality of life of patients. T2DM also increases the incidence of emotional diseases (Katon et al., 2005). In addition, some studies have shown that this disease can also have a significant impact on employment, reproduction and some other issues (Tunceli et al., 2005). Despite the fact that we have now developed many hypoglycemic agents, they have not been effective in stopping the progression of T2DM. Currently, this disease has become one of the major causes of mortality worldwide (Saeedi et al., 2020). T2DM-related medical expenses caused a heavy burden on the medical system and have a serious impact on the social economy (Peters et al., 2017; American Diabetes Association 2018; Dall et al., 2019). Thus, management of T2DM is still a research hotspot.

In China, traditional Chinese medicine (TCM) is widely used as an adjunctive treatment for T2DM, with Huang-Lian Jie-Du decoction (HLJDD) being one of the representative formulas. HLJDD was first recorded in *Emergency Formulas to Keep Up One*’*s Sleeve (Zhŏu Hòu Bèi Jí Fāng*), consisting of four herbs: Coptis chinensis Franch. Ranunculaceae (*huáng lián*), Scutellaria baicalensis Georgi. Lamiaceae (*huáng qín*), Phellodendron chinense Schneid. Rutaceae (*huáng băi*) and Gardenia jasminoides Ellis. Rubiaceae (*zhī zĭ*). According to the basic theory of TCM, HLJDD can relieve T2DM by clearing heat and resolving toxins and thus can have a therapeutic effect on T2DM. The main active ingredients in this formula, which were measured by HPLC, include geniposide, berberine, palmatine, etc (YANG et al., 2019). These ingredients were found to be effective in treating T2DM through a variety of mechanisms (Gu et al., 2010; Zhang et al., 2010; Li et al., 2019a; Li et al., 2019b; Mi et al., 2019; Sun et al., 2020a; Sun et al., 2020b; Wu et al., 2020). A series of clinical studies have been carried out on the treatment of T2DM with HLJDD, and promising results have been obtained. However, due to the limitations in study design, these studies were unable to provide conclusive evidence for HLJDD in treating T2DM.

The aim of systematic review is to provide an accurate presentation of this issue. Since systematic review plays an increasingly important role in health care decisions, it is critical to ensure its accuracy. Regardless of the certainty of evidence, accurate presentation can provide reliable guidance for clinical decision making. In 2018, two systematic-reviews of HLJDD for T2DM were published, but they both had serious methodological flaws (CHEN et al., 2018; GUO 2019). Some other clinical trials were carried out after the publication of these two meta-analyses. Given this situation, this study systematically summarized and evaluated the clinical evidence in treating T2DM with HLJDD by using evidence-based medicine methods, aiming to provide an up-to-date and accurate presentation of this issue for research and clinical practice.

## Methods

This study has been registered in advance on the website of Open Science Framework (OSF, https://osf.io/) with a registration number of DOI: 10.17605/OSF.IO/AJDU3. The registered website is: https://osf.io/ajdu3. In order to ensure the reliability of this research, we carried out this meta-analysis under the guidance of the latest *Cochrane Handbook for Systematic Reviews of Interventions version 6.0 (updated July 2019)* and methodological expectations for conduct, reporting and updating of systematic reviews of intervention (MECIR).

### Database and Search Strategies

Six databases including PubMed, embase, Cochrane Central Register of Controlled Trial (CENTRAL), China National Knowledge Infrastructure (CNKI), Wanfang Data Knowledge Service Platform and VIP information resource integration service platform (cqvip) were searched from inception to June 27, 2020 without language and publication status restrictions. Search strategies that combined controlled vocabulary and text words were developed. ClinicalTrials.gov, WHO International Clinical Trials Registry Platform (ICTRP) portal and Chinese Clinical Trial Registry (CHiCTR) were also searched to find out ongoing research. In addition, reference lists of reviews and meta-analyses were also searched. Detailed search strategies of bibliographic databases were provided in Supplementary Material S1.

### Inclusion and Exclusion Criteria

#### Type of Studies

Randomized controlled trials (RCTs) were included. Observational studies were not included due to potential high risk of bias and confounding. Observational studies include non-randomized controlled trial, controlled before-and-after study, interrupted time series study, historically controlled study, cohort study, case-control study, cross-sectional study, case series (uncontrolled longitudinal study).

#### Type of Participants

Adults (≥18 years old) with an established diagnosis of T2DM were included. Ideally, the diagnostic criteria should be reported in papers. If a study did not report diagnostic criteria specifically, we excluded it as a sensitivity analysis to explore its impact on the results. There was no restriction on other demographic factors of participants and settings.

#### Type of Interventions

HLJDD at any mode, dose, duration, or frequency of delivery was included. The composition of HLJDD cannot be modified. Considering the diversity of scenarios in the clinical application of HLJDD, we included metformin or no treatment as control groups in this study. Treatment group can be HLJDD alone or combination therapy of HLJDD and metformin. Co-interventions, if administrated in intervention group, should be the same in control group.

#### Type of Comparisons

To ensure clinical homogeneity, data analyses and presentation of results were carried out strictly based on the following comparisons:

Combination of HLJDD and metformin vs. metformin alone.

HLJDD alone vs. metformin.

HLJDD alone vs. no treatment.

#### Type of Outcome Measures

##### Primary Outcome

Glycated hemoglobin (HbA1c).

##### Secondary Outcomes

Fasting blood glucose (FBG).

Two-hour Postprandial glucose (2hPG).

Homeostasis model assessment of insulin resistance (HOMA-IR).

Body mass index (BMI).

Blood lipid profile: High Density Liptein Cholesterol (HDL-C), Low-Density Lipoprotein Cholesterol (LDL-C), Total Cholesterol (TC), Triglyceride (TG).

##### Safety Outcome

Adverse events.

### Data Collection and Analysis

#### Study Selection

Two evaluators screened citations independently by reading titles and abstracts. Full text of potentially qualified literature was obtained. Discrepancies were solved by consultation with a third author. If discrepancies still cannot be resolved after consultation, those articles would be classified as “Studies Awaiting Classification”. The list of studies excluded after reading full text and reason for exclusion were provided in Supplementary Material S2.

#### Data Extraction

A pre-specified template was constructed to collect the following data from studies: First author and year, Country, Study design, Diagnostic criteria, Age (treatment/control) (years), No. of Patients (treatment/control), female (treatment/control), Duration of T2DM (treatment/control) (year), Co-intervention, Treatment, Comparator, Run-in period, Duration of treatment, Follow-up, Funding, Baseline HbA1C (treatment/control) (%), Baseline BMI (treatment/control), Baseline FBG (mmol/L), Baseline 2hPG (mmol/L), Baseline HOMA-IR, Baseline TC (mmol/L), Baseline TG (mmol/L), Baseline HDL-C (mmol/L), Baseline LDL-C (mmol/L), HbA1C (treatment/control), BMI (treatment/control), post-intervention FBG (mmol/L), post-intervention 2hPG (mmol/L), HOMA-IR, post-intervention TC (mmol/L), post-intervention TG (mmol/L), post-intervention HDL-C (mmol/L), post-intervention LDL-C (mmol/L), comorbidity, adverse event. For studies with multiple publications, we combined these publications and used them in research. We contacted the authors for additional information by email if necessary. Data extraction was carried out by two authors independently, and discrepancies were solved by discussion.

#### Risk of Bias Assessment

In order to ensure the methodological reliability of this study, the risk of bias of the included studies was assessed by using the latest 2019 version 2 of the Cochrane risk-of-bias tool for randomized trials (RoB2) (JAC et al., 2019). Detailed guidance of assessment can be found on the website https://www.riskofbias.info/. The nature of the effect of interest was “intention-to-treat” effect. Discrepancies were solved by discussion with a third author, Hongyan Xie. We plotted “traffic light” plot and weighted bar plot by using the robvis package for R to show the risk of bias assessment results (McGuinness and Higgins 2020). Finally, in order to promote transparency of assessment, we provided supporting information in Supplementary Material S3.

### Data Synthesis

Data analysis was carried out according to *Cochrane Handbook for Systematic Reviews of Interventions* version 6.0 (updated August 2019). Meta package for R was used for data synthesis. Risk ratio (RR) with 95% confidence interval (CI) was used as an effect measure for dichotomous outcomes and mean difference (MD) with 95% CI was used for continuous outcomes (Borenstein et al., 2017; Deeks et al., 2019a; Deeks et al., 2019b). Chi^2^ test was used to test the heterogeneity among studies and a significant level of *p* < 0.1 was considered to be heterogeneous. *I* ^*2*^ statistics were used to quantify inconsistency across studies and *I* ^*2*^ greater than 50% may represent substantial heterogeneity. A random-effects model was used to pooled the studies if there was substantial heterogeneity. As a sensitivity analysis, we also used another statistical model (fixed or random) to calculate the effect size and compare the results of two models to explore the impact of the model on the results. Prediction intervals were not calculated as the number of included studies was less than ten [(Riley et al., 2011; Veroniki et al., 2019)]. If one outcome was considered inappropriate for data synthesis, we presented this result by narrative overview.

### Subgroup Analysis

Subgroup analyses were carried out according to these prespecified subgroup hypotheses (Oxman and Guyatt 1992; Sun et al., 2010):

duration of T2DM (≤5 years or >5 years).

Treatment duration (≥3 months or <3 months).

Age (>50 years old or ≤50 years old).

Baseline level (depending on data).

### Sensitivity Analysis

Sensitivity analyses were conducted to investigate the robustness of the results by re-calculating pooled estimates after omitting one study at a time and using another statistical model (random-effects model or fixed-effect model).

### Publication Bias

Publication bias was not carried out because of insufficient included studies.

### Certainty of Evidence

Certainty of evidence was evaluated by grading of recommendations assessment, development, and evaluation (GRADE) methodology (Guyatt et al., 2008). The evidence from RCT was initially rated as high quality and can be degraded for reasons including risk of bias, imprecision, inconsistency, indirectness and publication bias (Guyatt, et al., 2011a; Guyatt, et al., 2011b; Guyatt,et al., 2011c; Guyatt, et al., 2011d; Guyatt, et al., 2011e).

## Results

### Search Results

**FIGURE 1:**
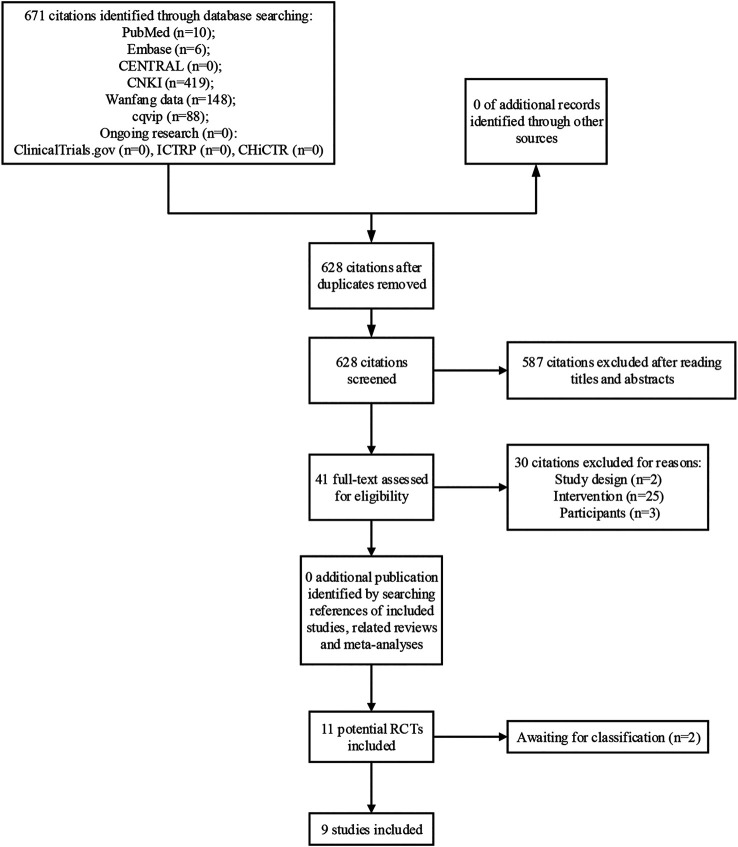
Study flow diagram.

A total of 671 citations were identified through database search. After reading titles and abstracts, 41 full-texts were obtained for further screening. Finally, 9 studies were included in meta-analysis and 2 studies were determined as awaiting classification due to lack of sufficient information (Weishan 2012; Wenjun and Pu 2013; Wenjun and Xingguo 2013; Jin 2015; Qiang 2018; Wen 2018; Xiang 2018; Jiajun 2019; Yajin 2020). A list of excluded studies by reading full-text was provided in Supplementary Material S2. The process of study selection was shown in Figure 1.

### Characteristics of Included Studies

Nine studies including 811 participants were included in this meta-analysis. All the studies were conducted in China, with a time span from 2005 to 2020. In terms of formula composition, one study did not provide detailed information about medicine, while other studies reported detailed composition of HLJDD (Wen 2018). None of these studies reported quality control or chemical analysis of HLJDD. As to diagnostic criteria, 7 studies used the WHO definition as a diagnostic criteria, 2 studies used Chinese guidelines, and 1 study did not report diagnostic criteria (Jin 2015). The impact of these two studies on the pooled effect size was explored in sensitivity analysis. One study compared the difference in efficacy between metformin, HLJDD and combination treatment of metformin plus HLJDD at the same time, and we split this study into part 1 and part 2 (Wenjun and Xingguo 2013). One study reported the effect of HLJDD compared to no intervention with both groups receiving lifestyle interventions (Weishan 2012). Characteristics of included studies was provided in Table 1.

**TABLE 1:** Characteristics of included studies.

Jin 2015	Wen (2018)	Jiajun (2019)	Xiang (2018)	Qiang (2018)	Yajin (2020)	Weishan (2012)	Part 2-Wenjun and Xingguo (2013)	Part 1-Wenjun and Xingguo (2013)	Wenjun and Pu (2013)	First author and year
China	China	China	China	China	China	China	China	China	China	Country
RCT	RCT	RCT	RCT	RCT	RCT	RCT	RCT	RCT	RCT	Study design
NR	China's guideline (2013)	WHO 2010	WHO 1999	WHO 1999	China's guideline (2017)	WHO 1999	WHO 1999	WHO 1999	WHO 1999	Diagnostic criteria
59.5 ± 7.6/60.9 ± 7.4	58.21 ± 5.94/57.52 ± 6.03	64.15 ± 7.52/64.71 ± 7.25	69.0 ± 4.6/69.2 ± 4.7	37.6 ± 5.2/38.3 ± 5.6	55.9 ± 7.5/55.5 ± 7.3	40.83 ± 6.05/41.23 ± 7.05	NR	NR	42 ± 16/40 ± 15	Age (treatment/control) (years)
73/73	50/50	45/45	52/52	33/32	54/54	30/30	18/18	18/18	33/33	No. of patients (treatment/control)
35/34	23/28	20/19	30/29	17/17	27/28	15/12	NR	NR	11/13	female (treatment/control)
3.2 ± 0.6/3.3 ± 0.8	6.27 ± 2.38/6.43 ± 2.75	4.48 ± 1.59/4.55 ± 1.67	4.3 ± 2.4/4.1 ± 2.5	10.8 ± 2.5/11.5 ± 2.1	4.7 ± 1.8/4.5 ± 1.6	NR	NR	NR	1.34 ± 1.51/1.72 ± 1.62	Duration of T2DM (treatment/control) (year)
lifestyle Intervention	NR	NR	NR	Lifestyle intervention	Lifestyle intervention	Lifestyle intervention	Lifestyle intervention	Lifestyle intervention	Lifestyle intervention	Co-intervention
HLJD (cortex phellodendri 6 g, scutellaria 10 g, coptis 15 g, gardenia jasminoides 10 g), bid	Insulin glargine + HLJD	metformin + HLJD (Cortex phellodendri 9 g, scutellaria baicalensis 9 g, coptis chinensis 12 g, gardenia jasminoides 12 g), 300 ml, bid	metformin + HLJD (cortex phellodendri 9 g, scutellaria baicalensis 9 g, coptis chinensis 12 g, gardenia jasminoides 12 g), 300 ml, bid	Metformin + HLJD (Cortex phellodendri 20 g, scutellaria 9 g, coptis 12 g, gardenia 6 g), bid, 150 ml/per	Alprostadil injection + HLJD (Cortex phellodendri 12 g, scutellaria 12 g, coptis 15 g, gardenia 9 g), bid, 200 ml/per	HLJD (Cortex phellodendri: Scutellaria:coptis:gardenia = 1:1:3:1)	metformin + HLJD (cortex phellodendri 9 g, scutellaria 9 g, coptis 12 g, gardenia 12 g), bid,150 ML/per	HLJD (cortex phellodendri 9 g, scutellaria 9 g, coptis 12 g, gardenia 12 g),bid,150 ML/per	metformin + HLJD (cortex phellodendri 6 g, scutellaria 10 g, coptis 15 g, gardenia 10 g), bid	Treatment
convention Treatment	The initial dose of insulin glargine was 0.2 IU/(kg · d)	Metformin dynamic dose, no more than 2 g per day	Metformin dynamic dose, no more than 2 g per day	Metformin; one tablets each time, bid	Alprostadil injection, 10μg, iv, qd	None	Metformin 500mg, tid	Metformin 500mg, tid	Metformin	Comparator
Nr	NR	NR	NR	NR	NR	NR	NR	NR	NR	Run-in period
24w	8w	12w	12w	12w	4w	16w	12w	12w	24w	Duration of treatment
Nr	NR	NR	NR	NR	NR	NR	NR	NR	NR	Follow-up
Nr	NR	NR	NR	NR	NR	NR	Shandong province TCM science and technology development plan project (no.: 2009–088)	Shandong province TCM science and technology development plan project (no.: 2009–088)	Shandong province TCM science and technology development plan project (no.: 2009–088)	Funding
Nr	9.26 ± 1.35/9.41 ± 1.28	5.82 ± 1.52/5.74 ± 1.44	5.6 ± 2.5/5.7 ± 2.4	NR	NR	8.8 ± 0.5/8.8 ± 0.5	8.22 ± 0.64/8.24 ± 0.69	8.13 ± 0.52/8.24 ± 0.69	9.6 ± 2.1/9.3 ± 2.5	Baseline HbA1C (treatment/control) (%)
Nr	NR	NR	NR	NR	NR	NR	NR	NR	26.6 ± 2.17/26.23 ± 2.02	Baseline BMI (treatment/control)
Nr	10.73 ± 2.61/10.98 ± 2.34	10.58 ± 3.53/10.24 ± 3.64	10.9 ± 3.5/10.2 ± 3.6	NR	NR	12.96 ± 1.81/12.64 ± 1.68	9.65 ± 0.85/9.79 ± 0.79	9.62 ± 0.66/9.79 ± 0.79	13.41 ± 2.14/13.35 ± 1.77	Baseline FBG (mmol/L)
Nr	15.82 ± 3.13/15.71 ± 2.89	NR	NR	NR	NR	NR	NR	NR	NR	Baseline 2hPG (mmol/L)
Nr	NR	NR	NR	NR	NR	2.48 ± 0.69/2.69 ± 0.66	2.3 ± 0.3/2.35 ± 0.34	2.34 ± 0.31/2.35 ± 0.34	2.65 ± 0.66/2.67 ± 0.63	Baseline HOMA-IR
Nr	4.65 ± 0.92/4.63 ± 0.87	6.72 ± 1.61/6.63 ± 1.54	1.8 ± 0.3/1.8 ± 0.4	NR	NR	NR	6.84 ± 0.55/6.79 ± 0.47	6.74 ± 0.37/6.79 ± 0.47	NR	Baseline TC (mmol/L)
Nr	2.27 ± 0.62/2.31 ± 0.58	1.81 ± 0.36/1.83 ± 0.34	6.6 ± 1.3/6.7 ± 1.1	NR	NR	2.34 ± 0.77/2.13 ± 0.45	3.27 ± 0.35/3.26 ± 0.37	3.24 ± 0.21/3.26 ± 0.37	NR	Baseline TG (mmol/L)
Nr	1.13 ± 0.36/1.12 ± 0.41	1.06 ± 0.25/1.03 ± 0.23	1.1 ± 0.3/1.0 ± 0.4	NR	NR	0.79 ± 0.19/0.74 ± 0.17	0.87 ± 0.41/0.89 ± 0.29	0.90 ± 0.25/0.89 ± 0.29	NR	Baseline HDL-C (mmol/L)
Nr	NR	4.58 ± 1.25/4.52 ± 1.20	4.6 ± 0.4/4.5 ± 0.5	NR	NR	3.64 ± 0.70/3.80 ± 0.64	3.71 ± 0.42/3.80 ± 0.34	3.67 ± 0.42/3.80 ± 0.34	NR	Baseline LDL-C (mmol/L)
Nr	6.23 ± 0.92/7.42 ± 1.04	3.75 ± 1.04/5.01 ± 1.38	3.8 ± 1.4/5.0 ± 2.1	5.88 ± 0.51/6.92 ± 0.63	NR	5.8 ± 0.3/6.1 ± 0.1	5.97 ± 0.54/6.87 ± 0.62	7.14 ± 0.54/6.87 ± 0.62	5.4 ± 1.7/6.4 ± 1.8	HbA1C (treatment/control)
Nr	NR	NR	NR	NR	NR	NR	NR	NR	22.38 ± 2.11/24.59 ± 2.18	BMI (treatment/control)
Nr	5.86 ± 1.29/6.82 ± 1.87	7.29 ± 2.47/8.52 ± 2.86	7.3 ± 1.2/8.6 ± 3.3	5.27 ± 0.54/6.91 ± 0.84	NR	5.85 ± 0.31/6.81 ± 0.32	5.28 ± 0.68/6.87 ± 0.91	7.09 ± 0.73/6.87 ± 0.91	5.21 ± 0.73/6.39 ± 1.15	Post-intervention FBG (mmol/L)
Nr	7.58 ± 2.03/8.94 ± 1.97	NR	NR	NR	NR	NR	NR	NR	NR	Post-intervention PBG (mmol/L)
Nr	NR	NR	NR	1.70 ± 0.22/2.12 ± 0.36	NR	1.99 ± 0.30/2.39 ± 0.38	1.71 ± 0.29/2.13 ± 0.42	2.11 ± 0.4/2.13 ± 0.42	1.71 ± 0.55/2.51 ± 0.48	HOMA-IR
Nr	3.91 ± 0.77/4.34 ± 0.83	4.32 ± 1.33/5.82 ± 1.41	4.2 ± 0.6/5.8 ± 0.8	6.00 ± 0.25/6.53 ± 0.44	NR	NR	5.37 ± 0.53/6.54 ± 0.47	6.02 ± 0.28/6.54 ± 0.47	NR	Post-intervention TC (mmol/L)
Nr	1.69 ± 0.73/2.11 ± 0.66	1.19 ± 0.25/1.68 ± 0.31	1.2 ± 0.5/1.7 ± 0.3	2.91 ± 0.23/3.15 ± 0.36	NR	1.04 ± 0.34/1.26 ± 0.44	2.00 ± 0.26/3.13 ± 0.37	2.96 ± 0.24/3.13 ± 0.37	NR	Post-intervention TG (mmol/L)
Nr	1.41 ± 0.40/1.25 ± 0.38	1.72 ± 0.37/1.35 ± 0.31	1.7 ± 0.3/1.3 ± 0.4	1.13 ± 0.14/0.95 ± 0.33	NR	1.39 ± 0.34/1.00 ± 0.18	1.02 ± 0.34/0.97 ± 0.34	1.02 ± 0.23/0.97 ± 0.34	NR	Post-intervention HDL-C (mmol/L)
Nr	NR	2.27 ± 0.88/3.91 ± 1.17	2.3 ± 0.4/3.9 ± 1.0	3.48 ± 0.39/3.64 ± 0.31	NR	2.67 ± 0.42/2.94 ± 0.49	3.06 ± 0.49/3.61 ± 0.34	3.49 ± 0.41/3.61 0.34	NR	Post-intervention LDL-C (mmol/L)
Nr	NR	NR	NR	NR	NR	NR	NR	NR	NR	Comorbidity
Nr	Hypoglycemia symptoms occurred in 1 case in the treatment group and 3 cases in the control group; 4 cases in the treatment group showed mild nausea and did not want to eat and drink without special treatment, and then gradually disappeared. There was no gastrointestinal reaction in the control group	NR	NR	NR	There were 1 case of headache, 3 cases of nausea and vomiting, 1 case of dizziness and 1 case of dry cough in the treatment group there were 2 cases of headache, 1 case of nausea and vomiting, 1 case of dizziness and 1 case of dry cough	No abnormality	NR	NR	NR	Adverse event
Nr	NR	NR	NR	NR	DN	NR	NR	NR	NR	Diabetes-related complication

### Risk of Bias Assessment

**FIGURE 2:**
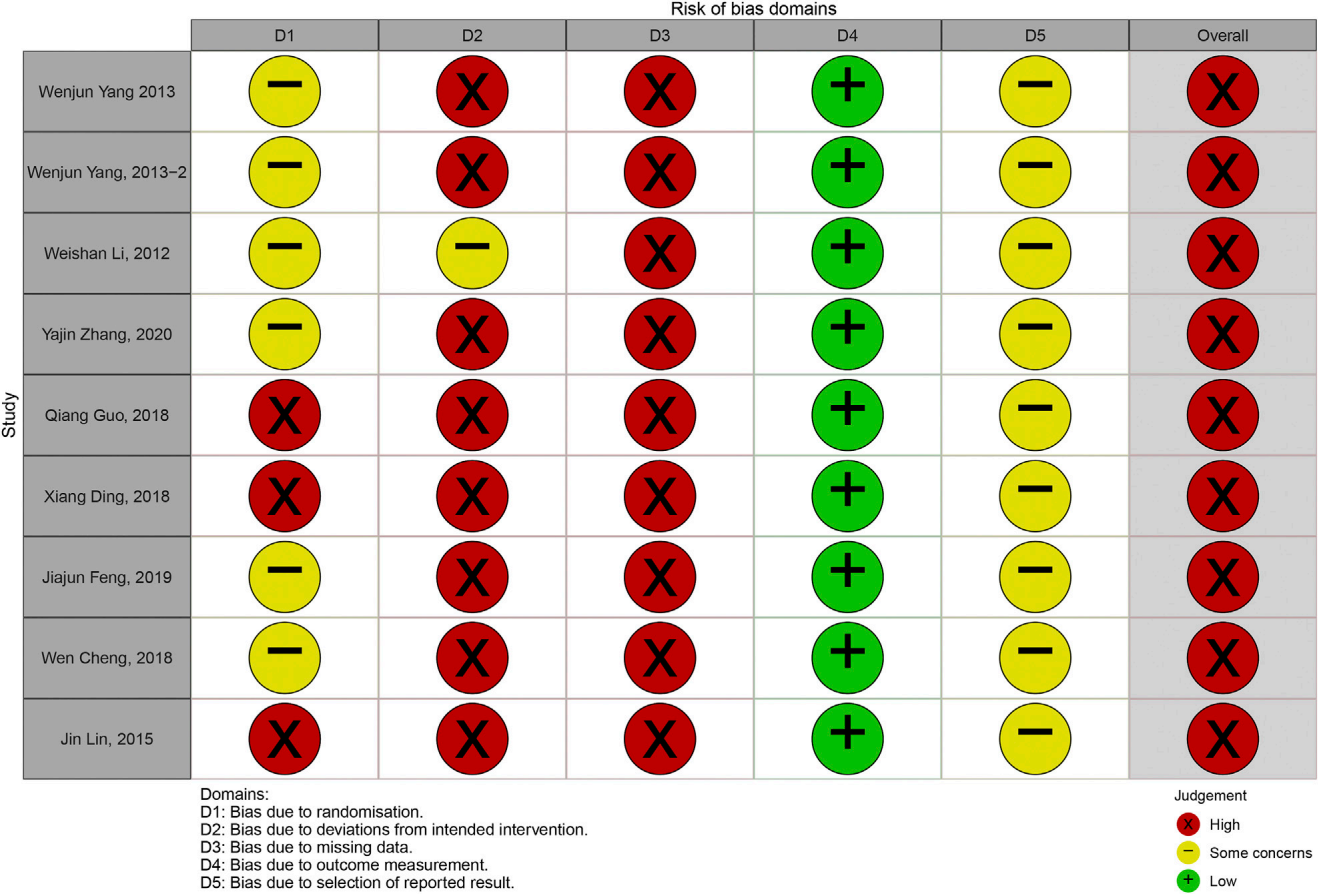
Risk of bias graph.

**FIGURE 3:**
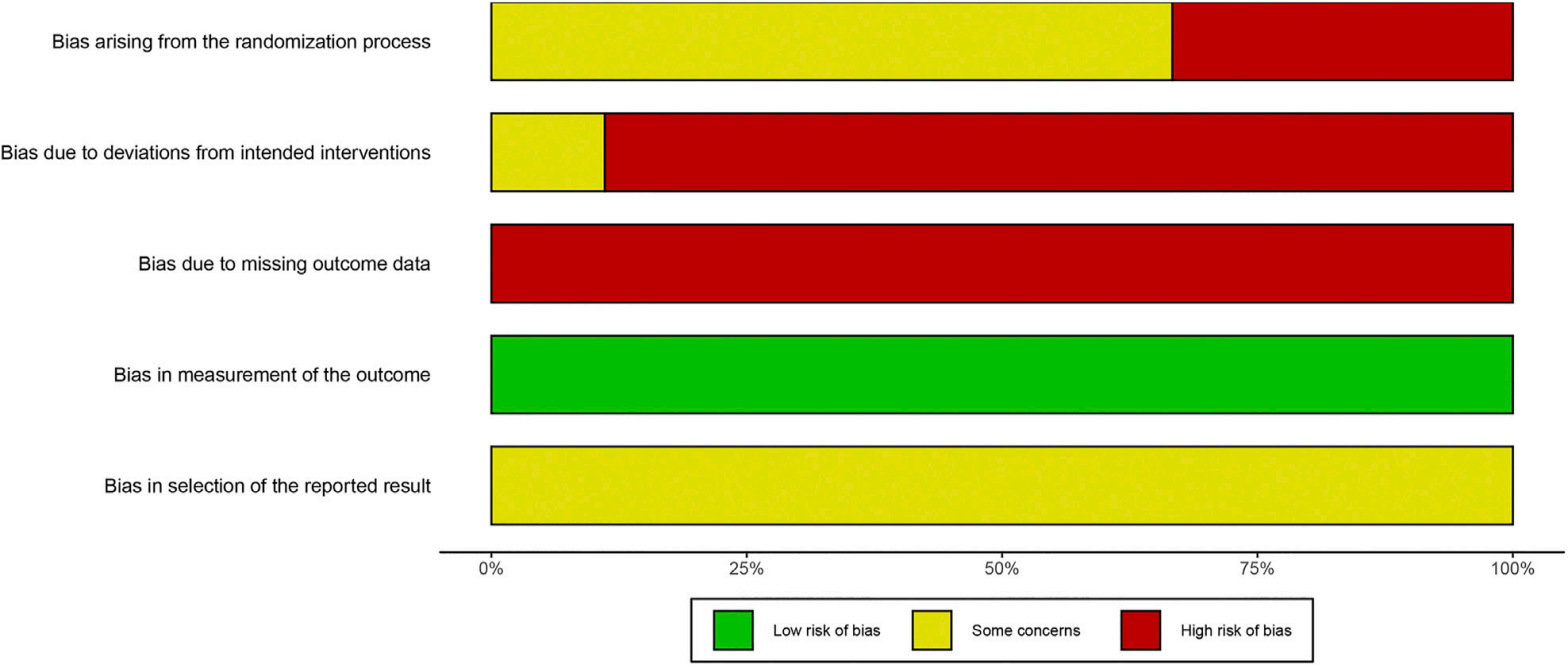
Risk of bias summary.

The overall judgements of risk of bias for all trials included in this study were high risk. The results of risk of bias were shown in Figures 2, 3. Details in risk of bias assessment were provided in supporting information (Supplementary Material S3).

### Glycated Hemoglobin

#### Combination of Huang-Lian Jie-Du Decoction and Metformin vs. Metformin Alone

**FIGURE 4:**
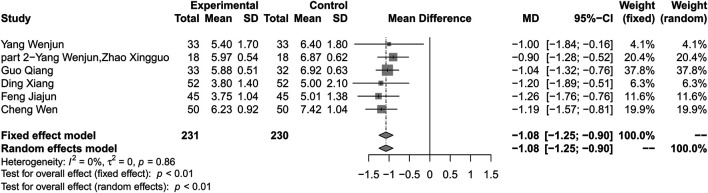
Forest plot for combination therapy of HLJDD and metformin compared with metformin on Hb1Ac.

Compared with metformin alone, combination treatment of HLJDD and metformin may result in a reduction in HbA1c (MD −1.08%; 95% CI −1.25 to −0.90; *p* < 0.01; *I* ^*2*^ = 0%; fixed effect model; 6 studies; 461 participants; low-certainty evidence) (Table 2, Figure 4) (Wenjun and Pu 2013; Wenjun and Xingguo 2013; Qiang 2018; Wen 2018; Xiang 2018; Jiajun 2019). Subgroup analyses according to different courses of T2DM, treatment durations, ages, baseline levels showed no significant difference between groups (*p* = 0.61, 0.51, 0.37, 0.42 respectively) (Supplementary Material S4). Sensitivity analysis by changing statistical model and omitting studies did not show significant changes in the pooled effect (Supplementary Material S5).

**TABLE 2:** Certainty of evidence.

Certainty assessment							№ of patients		Effect		Certainty	Importance
№ of studies	Study design	Risk of bias	Inconsistency	Indirectness	Imprecision	Other considerations	Combination therapy of HLJDD and metformin	Metformin	Relative (95% CI)	Absolute (95% CI)		
HbA1c												
6	Randomised trials	Very serious^a^	Not serious	Not serious	Not serious	None	231	230	-	MD 1.08% lower (1.25 lower to 0.9 lower)	⊕⊕○○ LOW	CRITICAL
FBG												
6	Randomised trials	Very serious^a^	Serious^b^	Not serious	Not serious	None	231	230	-	MD 1.41 mmol/L lower (1.64 higher to 1.18 higher)	⊕○○○ VERY LOW	CRITICAL
HOMA-IR												
3	Randomised trials	Very serious^a^	Serious^b^	Not serious	Very serious^c^	None	84	83	-	MD **0.53 lower** (0.76 lower to 0.31 lower)	⊕○○○ VERY LOW	IMPORTANT
HDL-C												
5	Randomised trials	Very serious^a^	Very serious^b^	Not serious	Very serious^c^	None	198	197	-	MD 0.24 mmol/L higher (0.12 higher to 0.37 higher)	⊕○○○ VERY LOW	IMPORTANT
LDL-C												
4	Randomised trials	Very serious^a^	Very serious^c^	Not serious	Very serious^c^	None	148	147	-	MD 0.98 mmol/L lower (1.73 higher to 0.22 higher)	⊕○○○ VERY LOW	IMPORTANT
TC												
5	Randomised trials	Very serious^a^	Very serious^b^	Not serious	Very serious^c^	None	198	197	-	MD 1.03 mmol/L lower (1.53 lower to 0.53 lower)	⊕○○○ VERY LOW	IMPORTANT
TC												
5	Randomized trials	Very serious^a^	Very serious^b^	Not serious	Serious^c^	None	198	197	-	MD 0.55 mmol/L lower (0.81 lower to 0.29 lower)	⊕○○○ VERY LOW	

a The overall quality of included studies was low.

b There are substantial differences in the results of point estimation and 95%CI.

c The 95% confidence interval is wide.

HLJDD, Huang-Lian Jie-Du decoction; T2DM, Type 2 diabetes mellitus; HbA1c, Glycated hemoglobin; FBG, Fasting blood glucose; HOMA-IR, Homeostasis model assessment of insulin resistance; HDL-C, High Density Liptein Cholesterol; LDL-C, Low-Density Lipoprotein Cholesterol; TC, Total Cholesterol; TG, Triglyceride; CI, Confidence interval; MD, Mean difference.

#### Huang-Lian Jie-Du Decoction Alone vs. Metformin

One study reported the effect of HLJDD alone on HbA1c compared with metformin and found that HLJDD could significantly decrease the level of HbA1c after three months of treatment but the effect was not as good as the metformin group (MD −0.17%; 95% CI −0.37 to 0.03; 36 participants) (Wenjun and Xingguo 2013).

#### Huang-Lian Jie-Du Decoction Alone vs. No Treatment

One study reported that HLJDD can reduce HbA1c compared with no intervention after four months treatment (MD −0.22%; 95% CI −0.42 to −0.02; 60 participants) (Weishan 2012).

### Fasting Blood Glucose

#### Combination of Huang-Lian Jie-Du Decoction and Metformin vs. Metformin Alone

**FIGURE 5:**
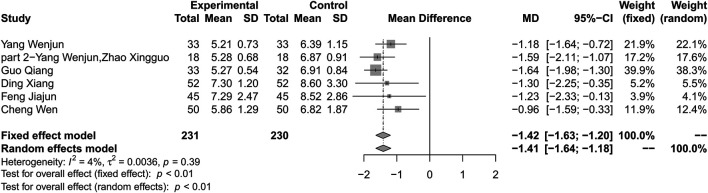
Forest plot for combination therapy of HLJDD and metformin compared with metformin on FBG.

Combination treatment of HLJDD and metformin may result in a reduction in FBG compared with metformin (MD −1.42; 95% CI −1.63 to −1.20; *p* < 0.01; *I* ^*2*^ = 4%; fixed effect model; 6 studies; 461 participants; very low-certainty evidence) (Table 2) (Figure 5) (Wenjun and Pu 2013; Wenjun and Xingguo 2013; Qiang 2018; Wen 2018; Xiang 2018; Jiajun 2019). Subgroup analyses according to different courses of T2DM, treatment durations, and ages showed no significant difference between groups (*p* for interaction = 0.18, 0.13 and 0.17 respectively) (Supplementary Material S6). Subgroup analysis based on baseline levels were not conducted due to limited information. Sensitivity analysis by changing statistical model and omitting studies did not show significant changes in the pooled effect (Supplementary Material S7).

#### Huang-Lian Jie-Du Decoction Alone vs. Metformin

One study did not found difference between HLJDD alone and metformin in FBG (MD 0.22; 95% CI −0.32 to 0.76; 36 participants) (Wenjun and Xingguo 2013).

#### Huang-Lian Jie-Du Decoction Alone vs. No Treatment

One study investigated the difference between HLJDD and no intervention and found that HLJDD plus lifestyle intervention could decrease FBG compared with lifestyle intervention (MD −0.96; 95% CI −1.12 to 0.8; 60 participants) (Weishan 2012).

### 2-h Postprandial Glucose

#### Combination of Huang-Lian Jie-Du Decoction and Metformin vs. Metformin Alone

One study reported that combination therapy of HLJDD and metformin may result in decrease in 2hPG compared with metformin alone (MD −1.36; 95% CI −2.14 to −0.58; 100 participants) (Wen 2018).

#### Huang-Lian Jie-Du Decoction Alone vs. Metformin or No Treatment

No study compared the effect of HLJDD with metformin or no intervention on 2hPG.

### Homeostasis Model Assessment of Insulin Resistance

#### Combination of Huang-Lian Jie-Du Decoction and Metformin vs. Metformin Alone

**FIGURE 6:**
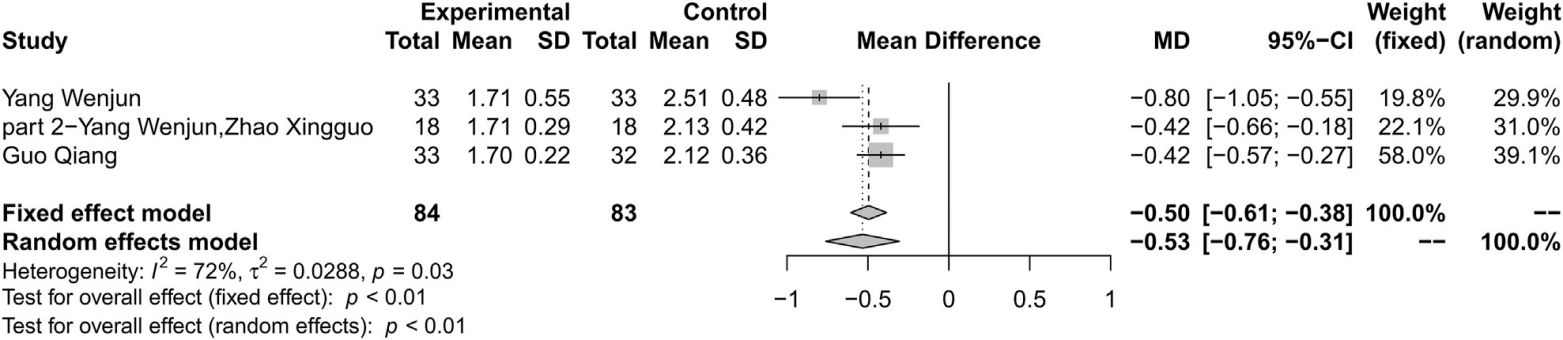
Forest plot for combination therapy of HLJDD and metformin compared with metformin on HOMA-IR.

Combination therapy of HLJDD and metformin could result in a reduction in HOMA-IR compared with metformin alone (MD –0.53; 95% CI –0.76 to –0.31; *p* < 0.01; *I* ^*2*^ = 72%; random-effects model; 3 studies; 167 participants; very low-certainty evidence) (Table 2) (Figure 6) (Wenjun and Pu 2013; Wenjun and Xingguo 2013; Qiang 2018). Subgroup analysis was not conducted. Sensitivity analysis by changing statistical model and omitting studies one by one did not show significant changes in the pooled effect (Supplementary Material S8).

#### Huang-Lian Jie-Du Decoction Alone vs. Metformin

One study did not found difference between HLJDD alone and metformin in HOMA-IR (MD –0.02; 95% CI –0.29 to 0.25; 36 participants) (Wenjun and Xingguo 2013).

#### Huang-Lian Jie-Du Decoction Alone vs. No Treatment

One study found that HLJDD plus no intervention could decrease HOMA-IR compared with no intervention (MD –0.40; 95% CI –0.57 to –0.23; 60 participants) (Weishan 2012).

### Body Mass Index

#### Combination of Huang-Lian Jie-Du Decoction and Metformin vs. Metformin Alone

No study report BMI for Combination of HLJDD and metformin vs. metformin alone.

#### Huang-Lian Jie-Du Decoction Alone vs. Metformin

One study found that there was an association between treatment by HLJDD and decrease of BMI compared with metformin alone (MD −0.22; 95% CI −0.42 to −0.02; 60 participants) (Wenjun and Pu 2013).

#### Huang-Lian Jie-Du Decoction Alone vs. No Treatment

No study report BMI for HLJDD alone vs. no treatment.

### High Density Liptein Cholesterol

#### Combination of Huang-Lian Jie-Du Decoction and Metformin vs. Metformin Alone

**FIGURE 7:**
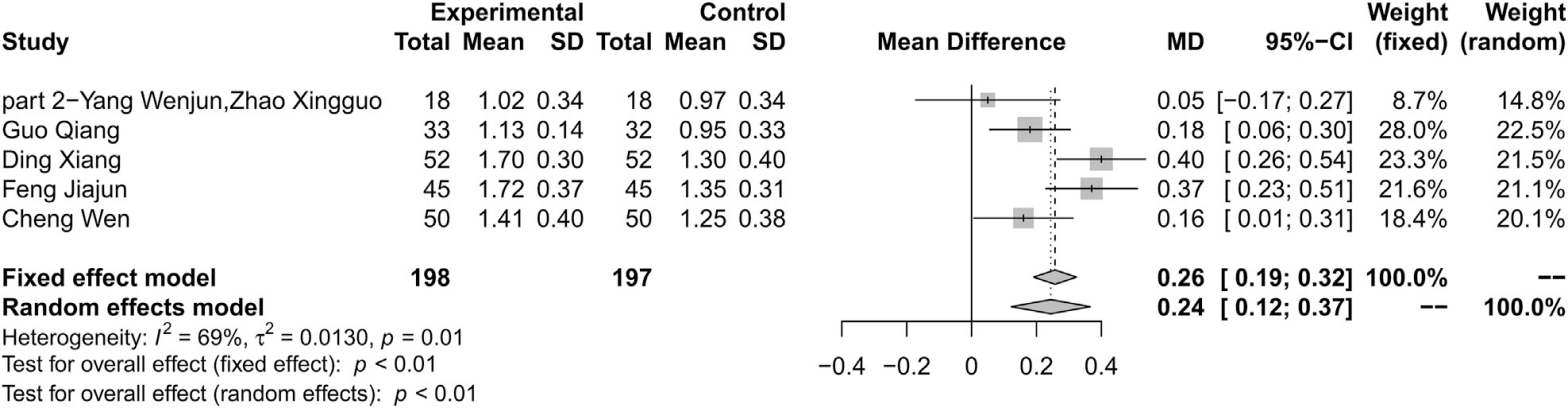
Forest plot for combination therapy of HLJDD and metformin compared with metformin on HDL-C.

Five studies reported HDL-C as outcome. Results showed that combination therapy can increase HDL-C level compared with metformin (MD 0.24; 95% CI 0.12 to 0.37; *p* < 0.01; *I* ^*2*^ = 69%; random-effects model; 5 studies; 395 participants; very low-certainty evidence) (Table 2) (Figure 7) (Wenjun and Xingguo 2013; Qiang 2018; Wen 2018; Xiang 2018; Jiajun 2019). Subgroup analyses by duration of T2DM, treatment duration, age and baseline level showed no significant difference in effect size (*p* for interaction = 0.94, 0.33, 0.17 and 0.88 respectively) (Supplementary Material S9). Sensitivity analysis by using changing statistical model and omitting studies one by one did not show significant changes in the pooled effect (Supplementary Material S10).

#### Huang-Lian Jie-Du Decoction Alone vs. Metformin

One study found that there was no significant difference between HLJDD and metformin in HDL-C (MD 0.05; 95% CI –0.14 to 0.24; 36 participants) (Wenjun and Xingguo 2013).

#### Huang-Lian Jie-Du Decoction Alone vs. No Treatment

One study reported that there was significant difference between HLJDD and no intervention in HDL-C (MD 0.39; 95% CI 0.25 to 0.53; 60 participants) (Weishan 2012).

### Low-Density Lipoprotein Cholesterol

#### Combination of Huang-Lian Jie-Du Decoction and Metformin vs. Metformin Alone

**FIGURE 8:**
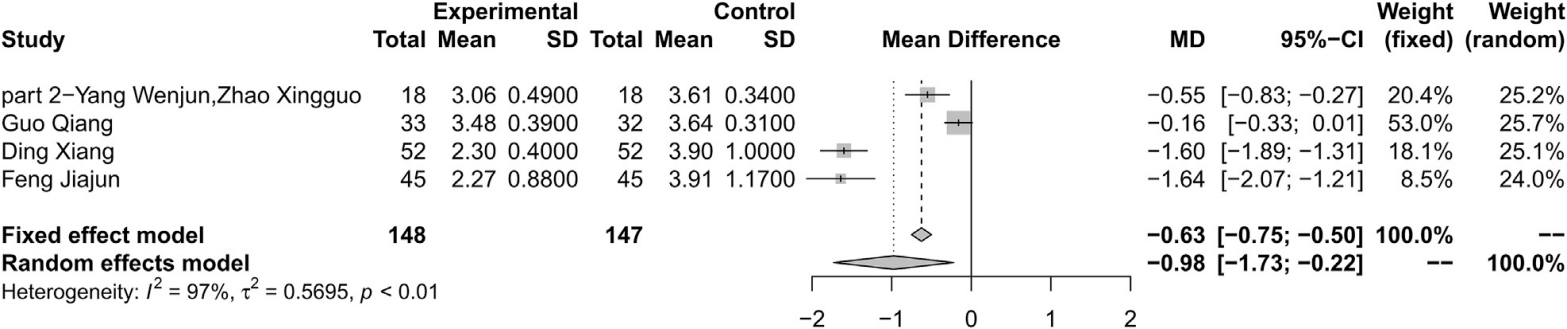
Forest plot for combination therapy of HLJDD and metformin compared with metformin on LDL-C.

Combination therapy of HLJDD and metformin may decrease the level of LDL-C compared with metformin alone (MD –0.98; 95% CI –1.73 to –0.22; *p* < 0.01; *I* ^*2*^ = 97%; random-effects model; 4 studies; 295 participants; very low-certainty evidence) (Table 2) (Figure 8) (Wenjun and Xingguo 2013; Qiang 2018; Xiang 2018; Jiajun 2019). Subgroup analyses by duration of T2DM showed no significant difference in effect size (*p* for interaction = 0.34). Subgroup analyses by age and baseline showed that there was significant difference in treatment effect (*p* < 0.01 for these subgroup analyses) (Supplementary Material S11). Sensitivity analysis showed that the result was not robust (Supplementary Material S12).

#### Huang-Lian Jie-Du Decoction Alone vs. Metformin

One study did not find significant difference between HLJDD alone and metformin in LDL-C (MD –0.12; 95% CI –0.37 to 0.13; 36 participants) (Wenjun and Xingguo 2013).

#### Huang-Lian Jie-Du Decoction Alone vs. No Treatment

One study found that HLJDD could decrease HOMA-IR compared with no intervention (MD –0.27; 95% CI –0.5 to –0.04; 60 participants) (Weishan 2012).

### Total Cholesterol

#### Combination of Huang-Lian Jie-Du Decoction and Metformin vs. Metformin Alone

**FIGURE 9:**
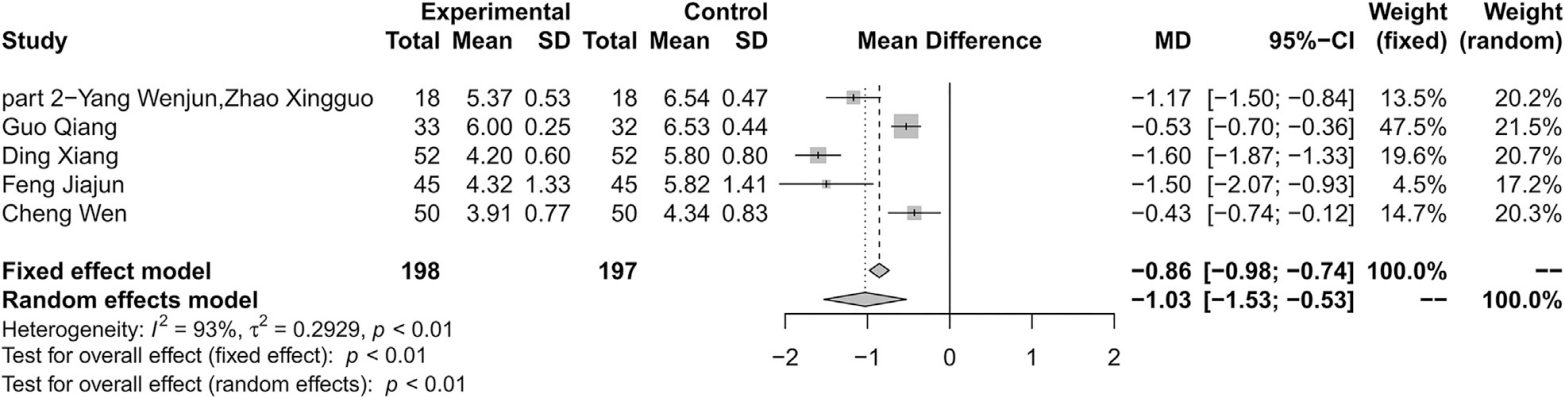
Forest plot for combination therapy of HLJDD and metformin compared with metformin on TC.

Combination therapy of HLJDD and metformin may decrease the level of TC compared with metformin alone (MD –1.03; 95% CI –1.53 to –0.53; *p* < 0.01; *I* ^*2*^ = 93%; random-effects model; 5 studies; 395 participants; very low-certainty evidence) (Table 2) (Figure 9) (Wenjun and Xingguo 2013; Qiang 2018; Wen 2018; Xiang 2018; Jiajun 2019). Subgroup analyses by duration of T2DM and age showed no significant difference in effect size (*p* for interaction = 0.96 and 0.14 respectively). Subgroup analyses by treatment duration and baseline showed that there was significant difference in treatment effect (*p* = 0.03 and *p* < 0.01 for these subgroup analyses) (Supplementary Material S13). Sensitivity analysis indicated that the result was robust (Supplementary Material S14).

#### Huang-Lian Jie-Du Decoction Alone vs. Metformin

One study found that there was significant difference between HLJDD alone and metformin in TC (MD –0.52; 95% CI –0.77 to 0.27; 36 participants) (Wenjun and Xingguo 2013).

#### Huang-Lian Jie-Du Decoction Alone vs. No Treatment

No study reported the difference between HLJDD and no intervention on TC as outcome.

### Triglyceride

#### Combination of Huang-Lian Jie-Du Decoction and Metformin vs. Metformin Alone

**FIGURE 10:**
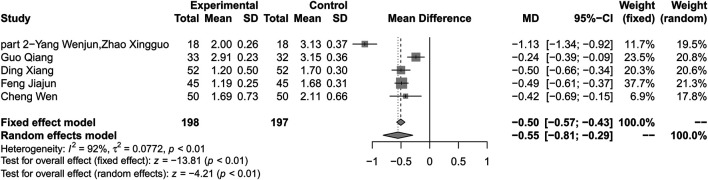
Forest plot for combination therapy of HLJDD and metformin compared with metformin on TG.

Combination therapy of HLJDD and metformin may decrease the level of TG compared with metformin alone (MD −0.55; 95% CI −0.81 to −0.29; *p* < 0.01; *I* ^*2*^ = 91%; random-effects model; 5 studies; 395 participants; very low-certainty evidence) (Table 2) (Figure 10) (Wenjun and Xingguo 2013; Qiang 2018; Wen 2018; Xiang 2018; Jiajun 2019). Subgroup analyses by age showed significant difference in effect size (*p* < 0.01). Subgroup analyses by duration of T2D, treatment duration and baseline level showed no significant difference in treatment effect (*p* = 0.44, 0.44 and 0.42 for these subgroup analyses) (Supplementary Material S15). Sensitivity analysis indicated that the result was not robust (Supplementary Material S16).

#### Huang-Lian Jie-Du Decoction Alone vs. Metformin

One study reported that there was no significant difference between HLJDD alone and metformin in TG (MD −0.17; 95% CI −0.37 to 0.03; 36 participants) (Wenjun and Xingguo 2013).

#### Huang-Lian Jie-Du Decoction Alone vs. No Treatment

One study reported that there was no significant difference between HLJDD and no intervention on TC (MD −0.22; 95% CI −0.42 to 0.02; 60 participants) (Weishan 2012).

### Adverse Events

Three studies reported adverse events as outcome. One study reported that no adverse event was observed (Weishan 2012). One study reported that there were 1 case of headache, 3 cases of nausea and vomiting, 1 case of dizziness and 1 case of dry cough in HLJDD group (Yajin 2020). One study reported 1 case of hypoglycemia and 4 cases of mild nausea and loss of appetite and then gradually disappeared (Wen 2018).

## Discussion

### Main Results of This Research

By using evidence-based methods, some important results were obtained from this study. A total of 671 relevant publications were searched, and eventually 9 studies were included in the quantitative analysis. By conducting a risk of bias assessment, we found that all studies suffered from high risk of bias. By pooling data, we found that the combination of HLJDD and metformin may reduce HbA1c, FBG, PBG, BMI, and HOMA-IR compared to metformin alone. In addition, we found that combination therapy was also associated with improved lipid metabolism, but the results were not robust. Evidence is insufficient regarding the use of HLJDD alone vs. metformin or vs. no intervention. Only 3 studies reported adverse events, so it is not yet sufficient to evaluate safety of HLJDD.

### Certainty of Evidence

We evaluated the certainty of evidence of this study through GRADEpro. As a result, it was found that the certainty of evidence regarding HLJDD combined with metformin was low. The degradation for the certainty of evidence was mainly due to high risk of bias, the inconsistency among studies and the imprecision of the findings. We did not evaluate the certainty of evidence for HLJDD alone vs. metformin alone or no intervention because there were too few research evidences.

### Risk of Bias

None of the trials included in this study implemented random allocation concealment and blinding methods well, which may lead to an exaggerated effect of the intervention. None of them use ITT analysis, which would have skewed the results in the direction of favoring HLJDD. Taking these points together, we think that the effect size obtained in this study may be overstated. The results of this study should be interpreted and applied with caution.

### Heterogeneity Among Studies

Statistical heterogeneity is a consequence of clinical or methodological heterogeneity, or both, among studies. By clearly defined the PICO for each comparison, the clinical heterogeneity was minimized. Due to the low methodological quality of the studies included in this study, as reflected by the risk of bias assessment results, this largely leads to the heterogeneity of the results. In addition, due to the small number of studies and sample size, we failed to find out more potential effect modifiers. This highlights the importance of strengthening the methodological control of future research.

### Publication Bias

Since fewer than 10 studies were included, we did not perform publication bias assessment in this study. The best way to evaluate whether there is publication bias is to compare published clinical trial report with registration information or study protocols. None of the trials included in this study were registered, and no studies protocol was available, so we were unable to assess the completeness of data. For these reasons, we cannot yet rule out the possibility of publication bias.

### Agreement and Disagreement With Other Studies

There are currently two systematic reviews about this issue, both of which suffered from serious methodological flaws [(CHEN et al., 2018; GUO 2019)]. In terms of literature search, this research conducted a more thorough data search and obtained some updated literature, which ensures that this research is up to date. In these two meta-analyses, modified HLJDD was also included in data analysis. In addition, some studies allowed researchers to adjust the composition of formula according to the patients' condition during the research. For these reasons, there were significant heterogeneity in formula composition among included studies. Combining these data in meta-analysis would lead to clinical heterogeneity and difficulties in the interpretation of results. In addition, it is not appropriate for authors to combine different comparisons when performing data analysis. The authors did not assess the certainty of evidence, which limited the application of results. Finally, the author confirmed the effects of HLJDD in T2DM, which in our view is inappropriate given the heterogeneity among studies, the high risk of bias and the potential publication bias.

### Implication for Clinical Practice and Future Research

This research was unable to draw any firm conclusion about the effects of HLJDD due to lack of high-quality evidence. Given the uncertainty of efficacy, clinicians should be cautious in recommending this prescription until more robust clinical studies are available. In addition, although there are some studies reporting adverse events, safety of HLJJD is still largely unknown, so clinicians and patients need to take into account that its potential risk have not been completely ruled out.

Based on the evidence now available, more high-quality studies evaluating the efficacy of HLJDD, particularly the effect of combination of HLJDD and metformin vs. metformin alone, are urgently needed. Based on the methodological evaluation in this research, the following points are suggested for special consideration in future research:

Use of proper placebo and masking in studies to evaluate placebo effects of HLJDD.

Register Studies Before They Begin to Reduce Selective Publication of Data

Use appropriate data analysis methods, such as ITT analysis.

Select Proper Sample Size Through Sample Size Estimation

Conduct follow-up visits to investigate long-term effect of HLJDD.

Improve the quality of reporting results by complying with the CONSORT statement.

### Limitation of This Research

Although we try our best to avoid bias during the study, some limitations still inevitably exist. The number of studies included in this study is small and the quality is low, which lead to the low certainty of evidence. Although we conducted subgroup analyses based on pre-defined subgroup hypotheses, the source of heterogeneity is still not fully identified. Due to the limited number of studies included, we were unable to perform meta-regression to further explore the source of heterogeneity. The cutoff points for age and duration are mainly based on related studies and more biological basis is needed. In addition, the included studies did not pay enough attention to the safety of HLJDD. In terms of effect on lipid metabolism, the results are not robust due to small number of included studies and inconsistency of results, which limit the clinical applicability of results.

## Conclusion

To sum up, this meta-analysis found that combination treatment of HLJDD and metformin may have an effect on T2DM but the evidence is very uncertain and more high-quality studies are needed to firmly establish the clinical efficacy and safety of HLJJD.

## Data Availability

The original contributions presented in the study are included in the article/Supplementary Material, further inquiries can be directed to the corresponding author.

## Abbreviations

2hPG, 2-h Postprandial glucose; CENTRAL, Cochrane Central Register of Controlled Trial; CHiCTR, Chinese Clinical Trial Registry; CI, confidence interval; CNKI, China National Knowledge Infrastructure; cqvip, VIP information resource integration service platform; FBG, Fasting blood glucose; GRADE, Grading of Recommendations Assessment, Development and Evaluation; HbA1c, Glycated hemoglobin; HDL-C, High Density Liptein Cholesterol; HLJDD, Huang-Lian Jie-Du decoction; HOMA-IR, Homeostasis model assessment of insulin resistance; ICTRP, WHO International Clinical Trials Registry Platform; LDL-C, Low-Density Lipoprotein Cholesterol; ROB2, version 2 of the Cochrane risk-of-bias tool for randomized trials; RR, Risk ratio; T2DM, Type 2 diabetes mellitus; TCM, traditional Chinese medicine; TC, Total Cholesterol; TG, Triglyceride.

## References

[B1] American Diabetes Association (2018). Economic Costs of Diabetes in the U.S. In 2017. Diabetes Care 41, 917–928. 10.2337/dci18-0007 29567642 PMC5911784

[B2] Borenstein M. Higgins J. P. T. Hedges L. V. Rothstein H. R. 2017. Basics of Meta-analysis:I2is Not an Absolute Measure of Heterogeneity. Res. Syn. Meth. 8:5–18. eng. 10.1002/jrsm.1230 28058794

[B3] Chen Y. Wu Q. Liu Y. Xiong S. (2018). Efficacy of Single Administration of Huanglian Jiedu Tang and Combined Administration with Basic Medicine for Type 2 Diabetes of Meta-Analysis. Chin. J. Exp. Traditional Med. Formulae (zhong guo Shi Yan fang ji Xue Za zhi) 24, 212–220. 10.13422/j.cnki.syfjx.20181041

[B4] Cho N. H. Shaw J. E. Karuranga S. Huang Y. da Rocha Fernandes J. D. Ohlrogge A. W. 2018. IDF Diabetes Atlas: Global Estimates of Diabetes Prevalence for 2017 and Projections for 2045. Diabetes Res. Clin. Pract. 138:271–281. eng. 10.1016/j.diabres.2018.02.023 29496507

[B5] Dall T. M. Yang W. Gillespie K. Mocarski M. Byrne E. Cintina I. 2019. The Economic Burden of Elevated Blood Glucose Levels in 2017: Diagnosed and Undiagnosed Diabetes, Gestational Diabetes Mellitus, and Prediabetes. Dia Care. 42:1661–1668. eng. 10.2337/dc18-1226 PMC6702607 30940641

[B6] Deeks J. J. Higgins J. P. T. Altman D. G. (2019a). “Chapter 10: Analysing Data and Undertaking Meta-Analyses,” in [Cochrane Handbook for Systematic Reviews of Interventions]. Editors Higgins J. P. T. Thomas J. Chandler J. Cumpston M. Li T. Page M. J. ([place Unknown]:Cochrane). Available from www.training.cochrane.org/handbook.

[B7] Deeks J. J. Higgins J. P. T. Altman D. G. (2019b). “Chapter 6: Analysing Data and Undertaking Meta-Analyses,” in [Cochrane Handbook for Systematic Reviews of Interventions]. Editors Higgins J. P. T. Thomas J. Chandler J. Cumpston M. Li T. Page M. J. ([place Unknown]:Cochrane). Available from www.training.cochrane.org/handbook.

[B8] Gu Y. Zhang Y. Shi X. Li X. Hong J. Chen J. (2010). Effect of Traditional Chinese Medicine Berberine on Type 2 Diabetes Based on Comprehensive Metabonomics. Talanta 81, 766–772. 10.1016/j.talanta.2010.01.015 20298851

[B9] Guo T. (2019). Efficacy of Single Administration of Huanglian Jiedu Tang and Combined Administration with Basic Medicine for Type 2 Diabetes of Meta-Analysis. New World of Diabetes (tang niao bing xin shi jie) 22, 53–54. CNKI:SUN:TNBX.0.2019-19-030

[B10] Guyatt G. H. Oxman A. D. Kunz R. Brozek J. Alonso-Coello P. Rind D. (2011a). GRADE Guidelines 6. Rating the Quality of Evidence-Imprecision. J. Clin. Epidemiol. 64, 1283–1293. 10.1016/j.jclinepi.2011.01.012 21839614

[B11] Guyatt G. H. Oxman A. D. Kunz R. Woodcock J. Brozek J. Helfand M. (2011b). GRADE Guidelines: 8. Rating the Quality of Evidence-Indirectness. J. Clin. Epidemiol. 64, 1303–1310. 10.1016/j.jclinepi.2011.04.014 21802903

[B12] Guyatt G. H. Oxman A. D. Kunz R. Woodcock J. Brozek J. Helfand M. (2011c). GRADE Guidelines: 7. Rating the Quality of Evidence-Inconsistency. J. Clin. Epidemiol. 64, 1294–1302. 10.1016/j.jclinepi.2011.03.017 21803546

[B13] Guyatt G. H. Oxman A. D. Montori V. Vist G. Kunz R. Brozek J. (2011d). GRADE Guidelines: 5. Rating the Quality of Evidence-Publication Bias. J. Clin. Epidemiol. 64, 1277–1282. 10.1016/j.jclinepi.2011.01.011 21802904

[B14] Guyatt G. H. Oxman A. D. Vist G. E. Kunz R. Falck-Ytter Y. Alonso-Coello P. (2008). GRADE: an Emerging Consensus on Rating Quality of Evidence and Strength of Recommendations. BMJ 336, 924–926. 10.1136/bmj.39489.470347.AD 18436948 PMC2335261

[B15] Guyatt G. H. Oxman A. D. Vist G. Kunz R. Brozek J. Alonso-Coello P. (2011e). GRADE Guidelines: 4. Rating the Quality of Evidence-Study Limitations (Risk of Bias). J. Clin. Epidemiol. 64, 407–415. 10.1016/j.jclinepi.2010.07.017 21247734

[B16] Jac S. Jelena S. Page M. J. Elbers R. G. Blencowe N. S. Boutron I. (2019). RoB 2: a Revised Tool for Assessing Risk of Bias in Randomised Trials. BMJ 366, l4898. 10.1136/bmj.l4898 31462531

[B17] Jiajun F. (2019). Clinical Effect of Huanglian Jiedu Decoction on Patients with Type 2 Diabetes Mellitus. J. China Prescription Drug (zhong guo chu fang yao) 17, 94–95. doi:CNKI:SUN:ZGCF.0.2019-04-065.

[B18] Jin L. (2015). Clinical Observation on 73 Cases of Obese Type 2 Diabetes Treated with Huanglian Jiedu Decoction. Chin. J. ethnomedicine ethnopharmacy (zhong guo min zu min jian Yi yao) 24, 70. doi:CNKI:SUN:MZMJ.0.2015-03-042.

[B19] Katon W. J. Rutter C. Simon G. Lin E. H. B. Ludman E. Ciechanowski P. (2005). The Association of Comorbid Depression with Mortality in Patients with Type 2 Diabetes. Diabetes Care 28, 2668–2672. 10.2337/diacare.28.11.2668 16249537

[B20] Li N. Li L. Wu H. Zhou H. (2019a). Antioxidative Property and Molecular Mechanisms Underlying Geniposide-Mediated Therapeutic Effects in Diabetes Mellitus and Cardiovascular Disease. Oxidative Med. Cell. longevity 2019, 1–20. 10.1155/2019/7480512 PMC6476013 31089416

[B21] Li N. Li L. Wu H. Zhou H. (2019b). Antioxidative Property and Molecular Mechanisms Underlying Geniposide-Mediated Therapeutic Effects in Diabetes Mellitus and Cardiovascular Disease. Oxidative Med. Cell. longevity 2019, 1–20. 10.1155/2019/7480512 PMC6476013 31089416

[B22] Li Y. Teng D. Shi X. Qin G. Qin Y. Quan H. 2020. Prevalence of Diabetes Recorded in Mainland China Using 2018 Diagnostic Criteria from the American Diabetes Association: National Cross Sectional Study. BMJ. 369:m997. eng. 10.1136/bmj.m997 32345662 PMC7186854

[B23] Magliano D. J. Islam R. M. Barr E. L. M. Gregg E. W. Pavkov M. E. Harding J. L. (2019). Trends in Incidence of Total or Type 2 Diabetes: Systematic Review. BMJ 366, l5003. 10.1136/bmj.l5003 31511236 PMC6737490

[B24] McGuinness L. A. Higgins J. P. T. (2020). Risk‐of‐bias VISualization (Robvis): An R Package and Shiny Web App for Visualizing Risk‐of‐bias Assessments. Res. Syn Meth 12, 55–61. 10.1002/jrsm.1411 32336025

[B25] Mi J. He W. Lv J. Zhuang K. Huang H. Quan S. (2019). Effect of Berberine on the HPA-axis Pathway and Skeletal Muscle GLUT4 in Type 2 Diabetes Mellitus Rats. Dmso 12, 1717–1725. 10.2147/DMSO.S211188 PMC6731988 31564939

[B26] Oxman A. D. Guyatt G. H. (1992). A Consumer's Guide to Subgroup Analyses. Ann. Intern. Med. 116:78–84. eng. 10.7326/0003-4819-116-1-78 1530753

[B27] Peters M. L. Huisman E. L. Schoonen M. Wolffenbuttel B. H. R. (2017). The Current Total Economic Burden of Diabetes Mellitus in the Netherlands. Netherlands: The Netherlands Journal of Medicine. [publisher unknown]. eng. 28956787

[B28] Qiang G. (2018). Psychological Monthly (xin li yue kan). The Weishan, 2012 is a dissertation and the shool name is Shandong University of Traditional Chinese Medicine.

[B29] Riley R. D. Higgins J. P. T. Deeks J. J. (2011). Interpretation of Random Effects Meta-Analyses. BMJ. 342:d549. eng. 10.1136/bmj.d549 21310794

[B30] Saeedi P. Salpea P. Karuranga S. Petersohn I. Malanda B. Gregg E. W. 2020. Mortality Attributable to Diabetes in 20-79 Years Old Adults, 2019 Estimates: Results from the International Diabetes Federation Diabetes Atlas, 9th Edition, 162(th) Edition. Diabetes Res. Clin. Pract. 162:108086. eng. 10.1016/j.diabres.2020.108086 32068099

[B31] Sun B. Jia X. Yang F. Ren G. Wu X. (2021a). CREB-mediated Generation and Neuronal Growth Regulates the Behavioral Improvement of Geniposide in Diabetes-Associated Depression Mouse Model. Neurosci. Res. 165, 38–44. 10.1016/j.neures.2020.05.003 32428538

[B32] Sun B. Jia X. Yang F. Ren G. Wu X. (2021b). CREB-mediated Generation and Neuronal Growth Regulates the Behavioral Improvement of Geniposide in Diabetes-Associated Depression Mouse Model. Neurosci. Res. 165, 38–44. 10.1016/j.neures.2020.05.003 32428538

[B33] Sun X. Briel M. Walter S. D. Guyatt G. H. 2010. Is a Subgroup Effect Believable? Updating Criteria to Evaluate the Credibility of Subgroup Analyses. BMJ. 340:c117. eng. 10.1136/bmj.c117 20354011

[B34] Tunceli K. Bradley C. J. Nerenz D. Williams L. K. Pladevall M. Elston Lafata J. (2005). The Impact of Diabetes on Employment and Work Productivity. Diabetes Care 28, 2662–2667. 10.2337/diacare.28.11.2662 16249536

[B35] Veroniki A. A. Jackson D. Bender R. Kuss O. Langan D. Higgins J. P. T. 2019. Methods to Calculate Uncertainty in the Estimated Overall Effect Size from a Random‐effects Meta‐analysis. Res. Syn Meth. 10:23–43. eng. 10.1002/jrsm.1319 30129707

[B36] Weishan L. (2012). The Clinical Observation of Huanglian Jiedu Decoction in the Treatment of Diabetes Combined Carotid Atherosclerosis. [MD] . [place unknown]: China, Shandong Shandong University of Traditional Chinese Medicine.

[B37] Wen C. (2018). Therapeutic Effect of Huanglian Jiedu Decoction Combined with Glargine Insulin on Glucose and Lipid Metabolism and Coagulation State in Patients with First-Episode T2DM. Cardiovasc. Dis. J. integrated traditional (zhong Xi Yi jie he xin Xue guan bing) 6, 160–161. 10.16282/j.cnki.cn11-9336/r.2018.36.121

[B38] Wenjun Y. Pu W. (2013). Research on the Intervention of Huanglian Jiedu Decoction on Obese Patients with Type 2 Diabetes. Shandong J. Traditional Chin. Med. (shan dong zhong Yi Za zhi) 32, 535–537.

[B39] Wenjun Y. Xingguo Z. (2013). Clinical Observation of Huanglian Jiedu Decoction Combined with Metformin in the Treatment of Type 2 Diabetes. Natl. Med. Front. China (zhong guo Yi yao qian yan) 8, 41–42. 10.3969/j.issn.1673-5552.2013.09.0024

[B40] Wu Y. S. Li Z. M. Chen Y. T. Dai S. J. Zhou X. J. Yang Y. X. (2020). Berberine Improves Inflammatory Responses of Diabetes Mellitus in Zucker Diabetic Fatty Rats and Insulin-Resistant HepG2 Cells through the PPM1B Pathway. J. Immunol. Res. 2020, 1–32. 10.1155/2020/2141508 PMC7450322 32908938

[B41] Xiang D. (2018). Clinical Effect of Traditional Chinese Medicine Huanglian Jiedu Decoction in the Treatment of Type 2 Diabetes. China J. Pharmaceutical Economics (zhong guo yao Wu jing ji xue) 13, 62–65. 10.12010/j.issn.1673-5846.2018.08.018

[B42] Yajin Z. (2020). Effects of Alprostadil and Huanglian Jiedu Decoction on Renal Function and Inflammatory Factors in Patients with Diabetic Nephropathy. Chronic Pathematology J.(man xing bing xue za zhi) 21, 418–420. 10.16440/j.cnki.1674-8166.2020.03.037

[B43] Yang L. Yuan Z. Ji P. Zhang X. Hua Y. Wei Y. (2019). Determination of 13 Active Components in Huanglian Jiedu Decoction by HPLC and Screening of its Effective Fraction. Chin. Traditional Herbal Drugs 50, 3794–3801. doi:CNKI:SUN:ZCYO.0.2019-16-009.

[B44] Zhang H. Wei J. Xue R. Wu J.-D. Zhao W. Wang Z.-Z. (2010). Berberine Lowers Blood Glucose in Type 2 Diabetes Mellitus Patients through Increasing Insulin Receptor Expression. Metabolism 59, 285–292. 10.1016/j.metabol.2009.07.029 19800084

