# Supplementary figures and images for: Effect of Huang-Lian Jie-Du Decoction on Glucose and Lipid Metabolism in Type 2 Diabetes Mellitus: A Systematic Review and Meta-Analysis
Source: Front Pharmacol. 2021 Apr 29;12:648861. doi: 10.3389/fphar.2021.648861 (PMC8117159; doi:10.3389/fphar.2021.648861)

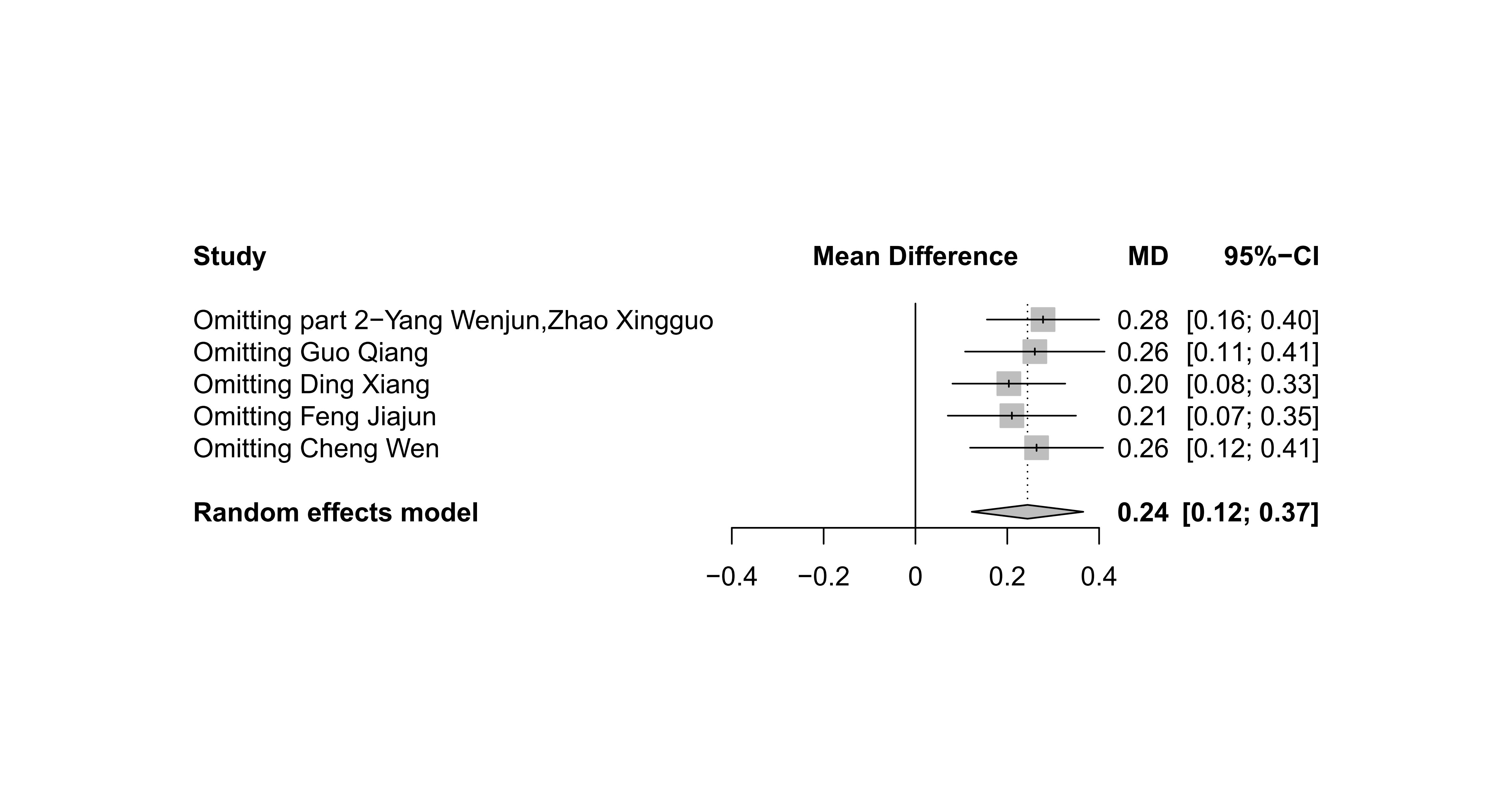

Supplement: Supplementary file 1 [file Image6.TIF]

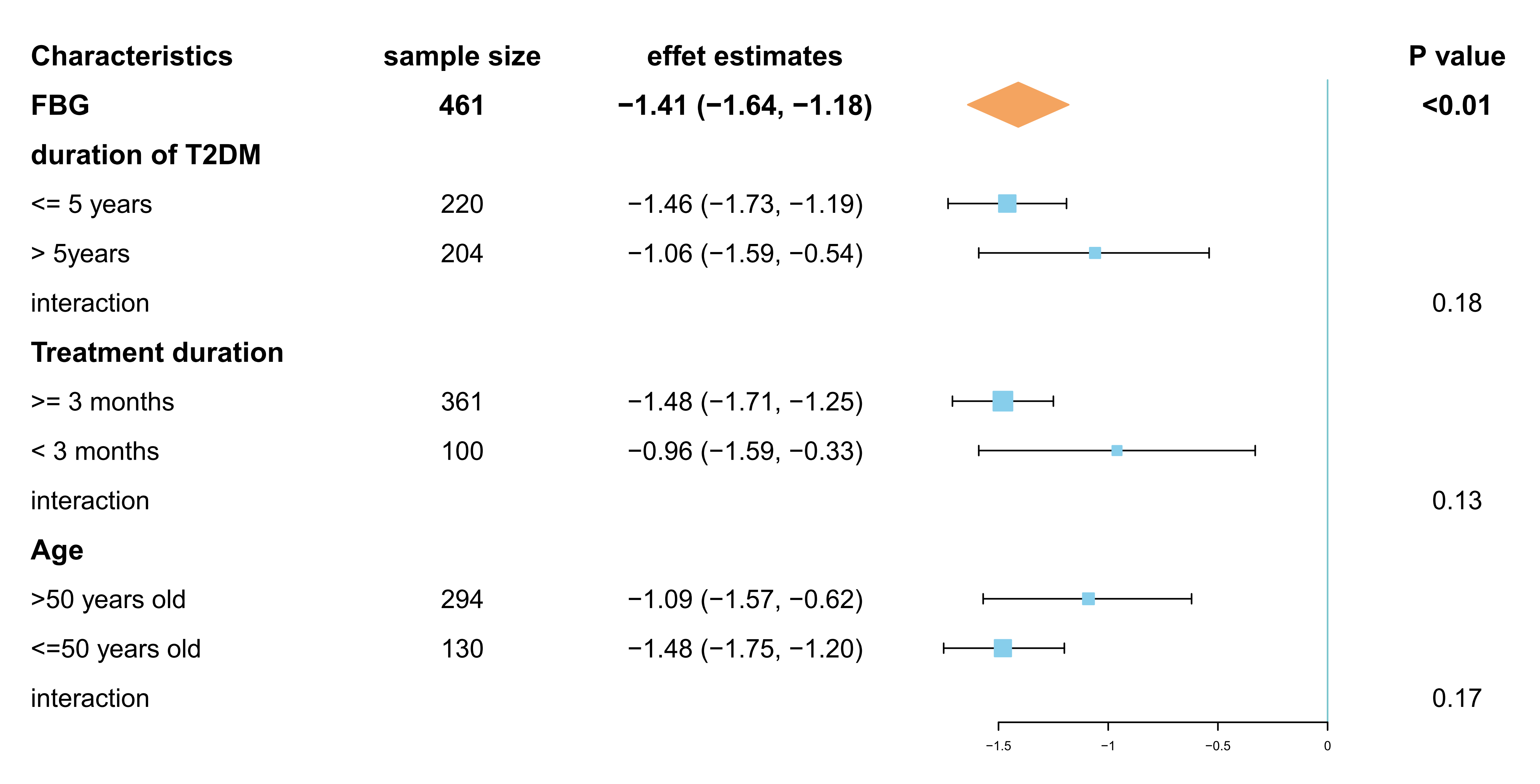

Supplement: Supplementary file 2 [file Image3.TIF]

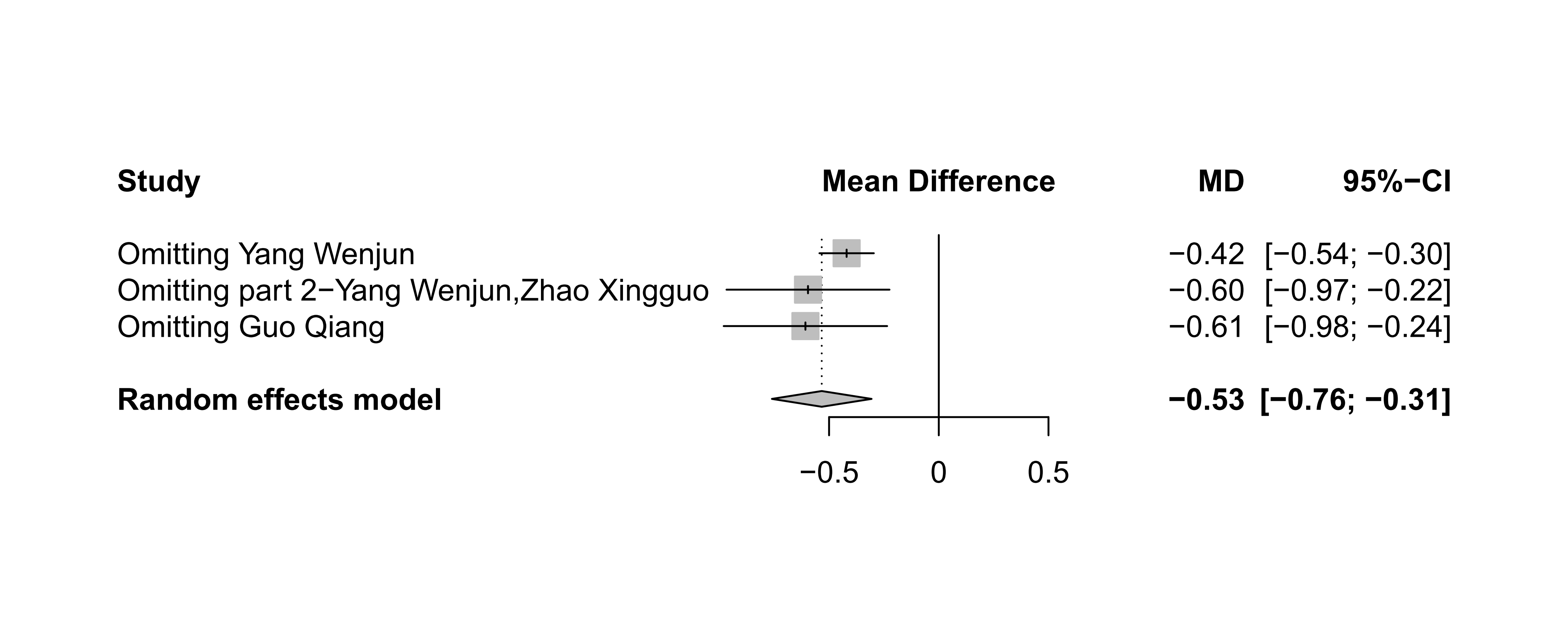

Supplement: Supplementary file 3 [file Image4.TIF]

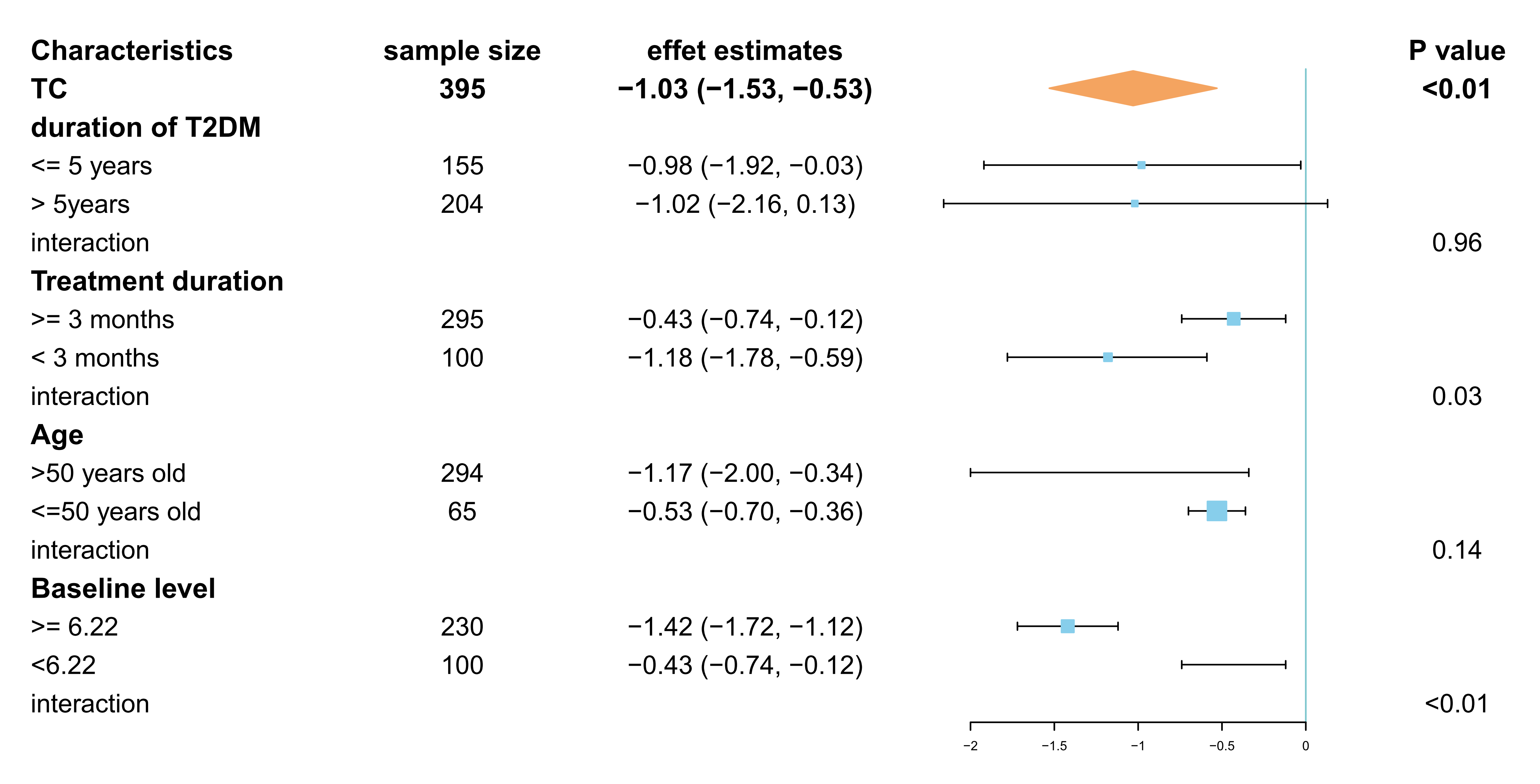

Supplement: Supplementary file 4 [file Image9.TIF]

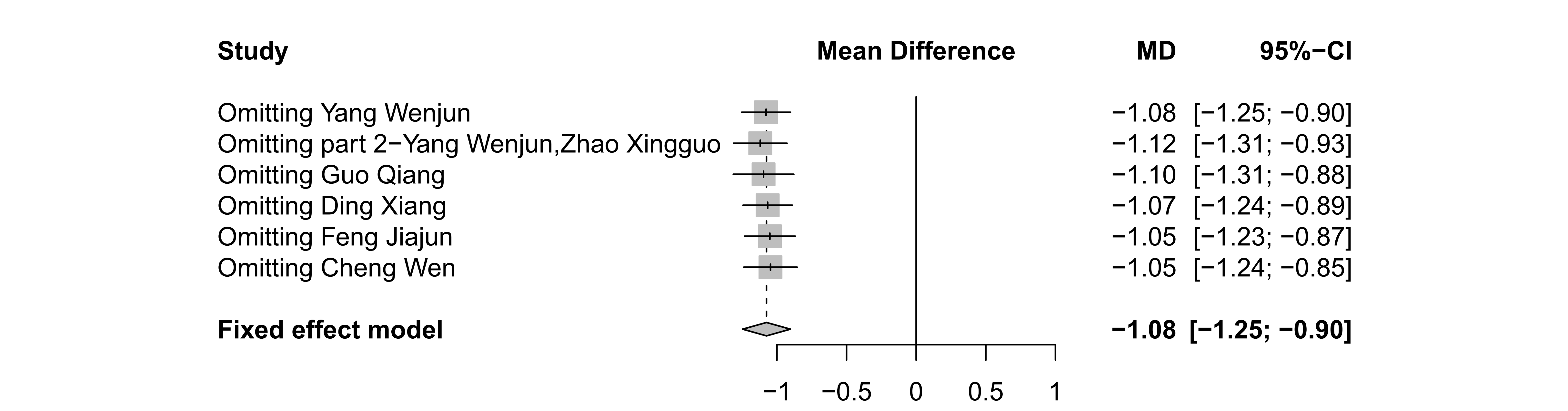

Supplement: Supplementary file 6 [file Image2.TIF]

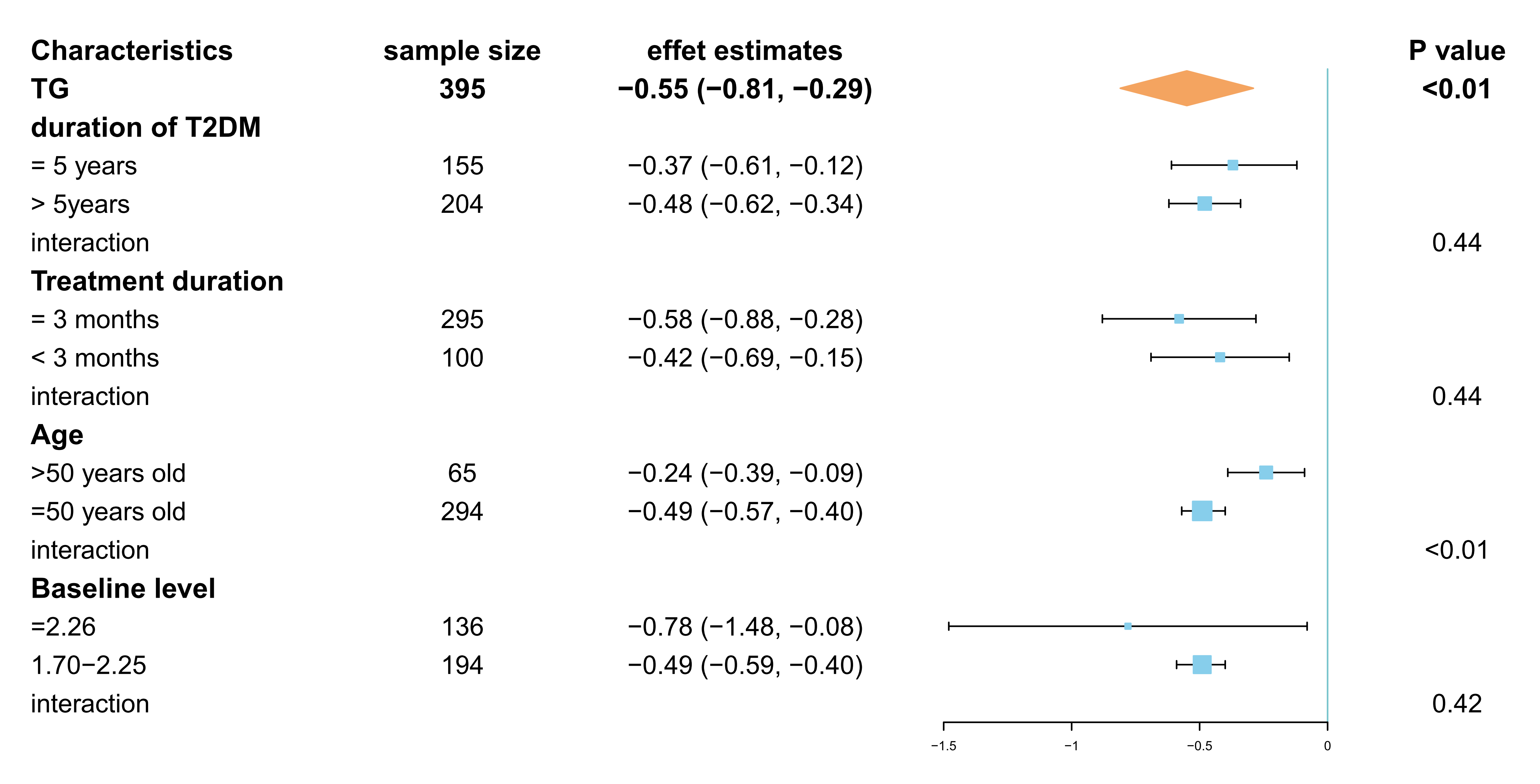

Supplement: Supplementary file 7 [file Image11.TIF]

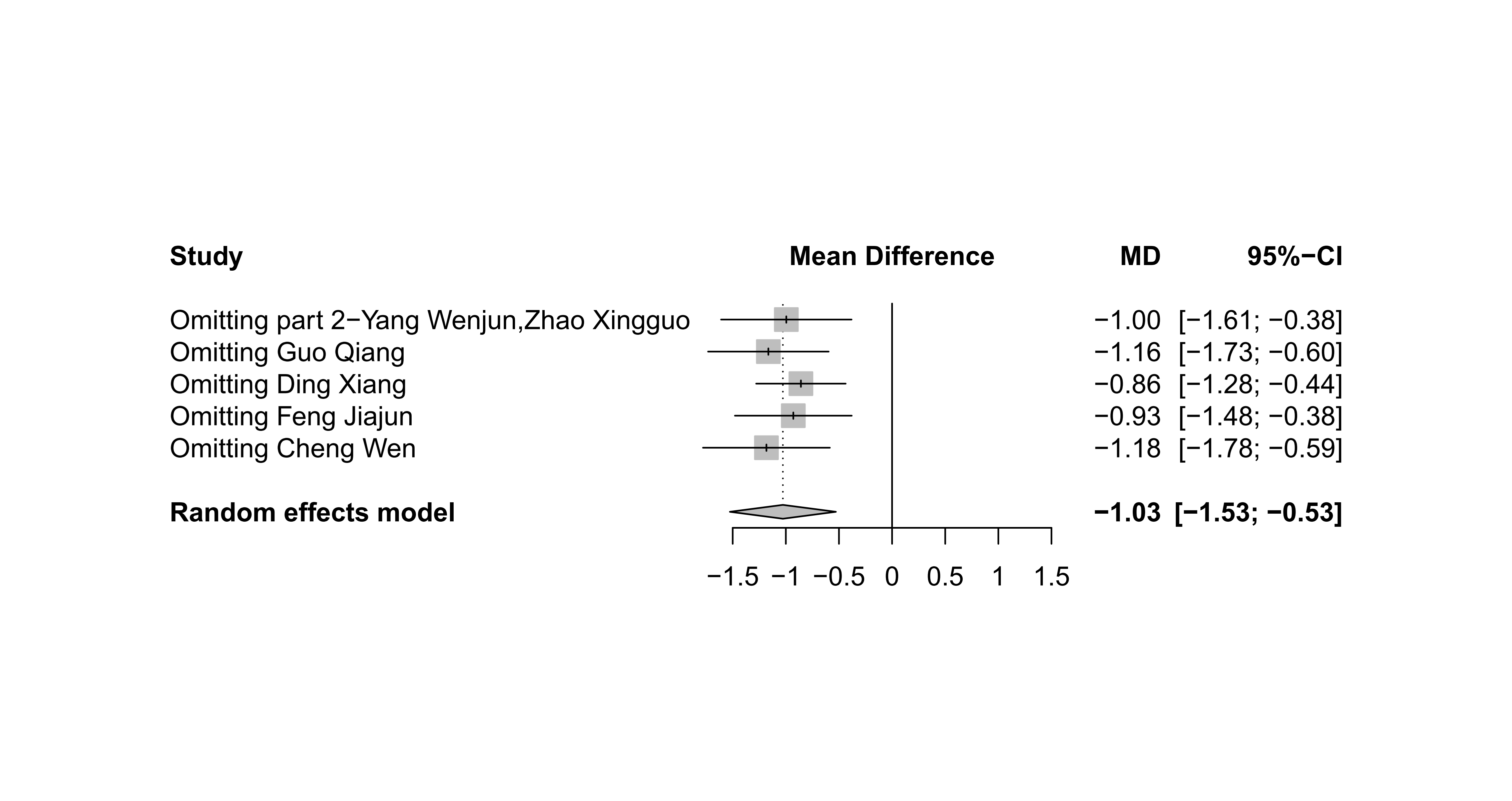

Supplement: Supplementary file 9 [file Image10.TIF]

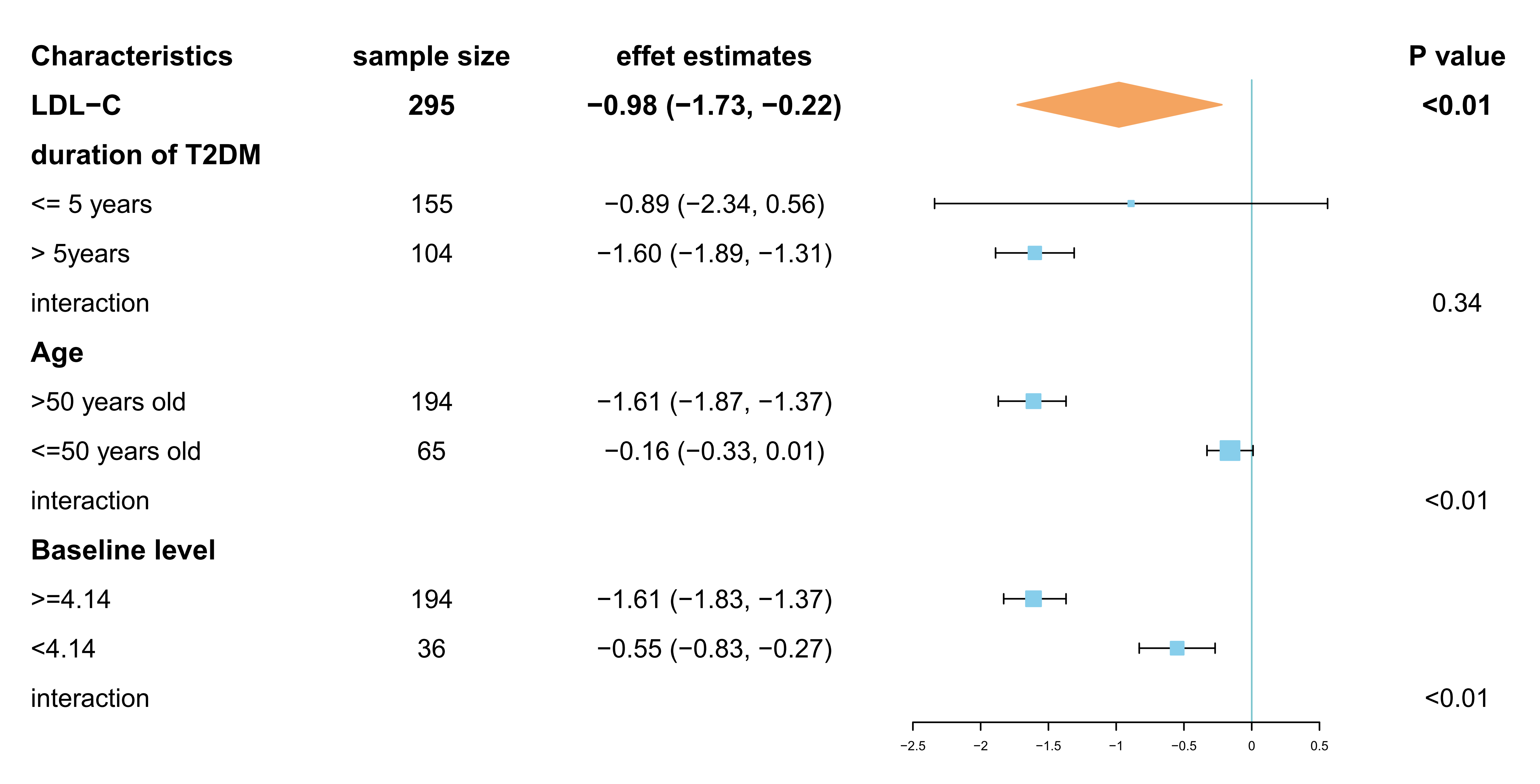

Supplement: Supplementary file 10 [file Image7.TIF]

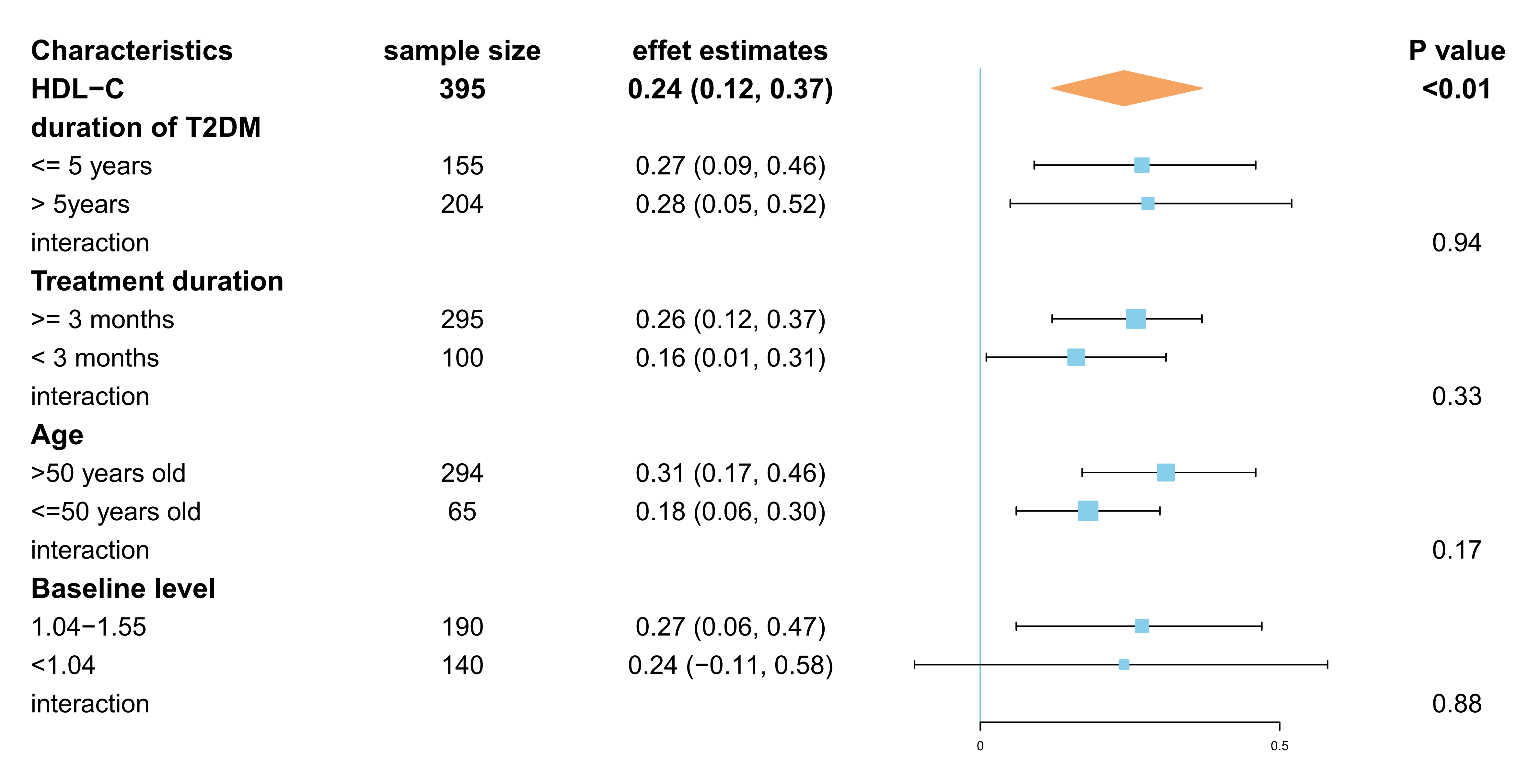

Supplement: Supplementary file 13 [file Image5.TIF]

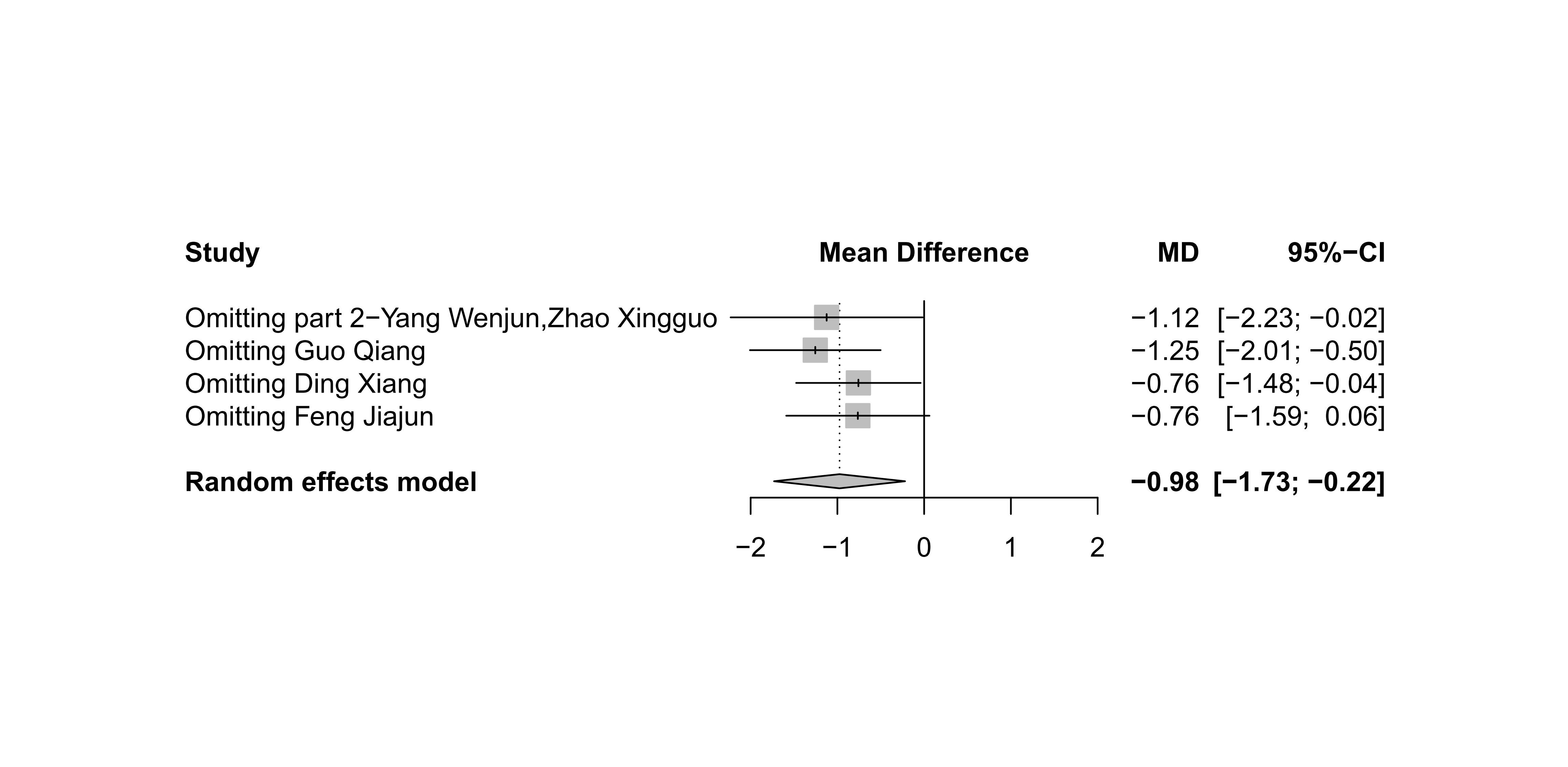

Supplement: Supplementary file 14 [file Image12.TIF]
