# Supplementary material for: Effect of Huang-Lian Jie-Du Decoction on Glucose and Lipid Metabolism in Type 2 Diabetes Mellitus: A Systematic Review and Meta-Analysis
Source: Front Pharmacol. 2021 Apr 29;12:648861. doi: 10.3389/fphar.2021.648861 (PMC8117159; doi:10.3389/fphar.2021.648861)
Supplement: Supplementary file 5 [file DataSheet3.docx]

1. **Wenjun Yang, 2013**

**Bias arising from the randomization process**

| Signaling question | Response | Supporting information |
| --- | --- | --- |
| 1.1 Was the allocation sequence random? | NI | The author did not elaborate on what random method was used. |
| 1.2 Was the allocation sequence concealed until participants were enrolled and assigned to interventions? | NI | The author didn't mention whether the allocation sequence was concealed. |
| 1.3 Did baseline differences between intervention groups suggest a problem with the randomization process? | PN | The two groups were comparable. |

**bias due to deviations from the intended interventions (effect of assignment to intervention)**

| Signaling question | Response | Supporting information |
| --- | --- | --- |
| 2.1. Were participants aware of their assigned intervention during the trial? | Y | Since there is no placebo control, it is easy for everyone to know the intervention. |
| 2.2. Were carers and people delivering the interventions aware of participants' assigned intervention during the trial? | Y | Since there is no placebo control, it is easy for everyone to know the intervention. |
| 2.3. If Y/PY/NI to 2.1 or 2.2: Were there deviations from the intended intervention that arose because of the trial context? | NI | No enough information. |
| 2.4 If Y/PY to 2.3: Were these deviations likely to have affected the outcome? | NA | - |
| 2.5. If Y/PY/NI to 2.4: Were these deviations from intended intervention balanced between groups? | NA | - |
| 2.6 Was an appropriate analysis used to estimate the effect of assignment to intervention? | N | ITT analysis was not used. |
| 2.7 If N/PN/NI to 2.6: Was there potential for a substantial impact (on the result) of the failure to analyses participants in the group to which they were randomized? | NI | No enough information. |

**Bias due to missing outcome data**

| Signaling question | Response | Supporting information |
| --- | --- | --- |
| 3.1 Were data for this outcome available for all, or nearly all, participants randomized? | NI | Since we don’t know the number of randomized patients, we cannot tell whether all patients’ data are available. |
| 3.2 If N/PN/NI to 3.1: Is there evidence that the result was not biased by missing outcome data? | PN | No enough information. |
| 3.3 If N/PN to 3.2: Could missingness in the outcome depend on its true value? | NI | No enough information. |
| 3.4 If Y/PY/NI to 3.3: Is it likely that missingness in the outcome depended on its true value? | NI | No enough information. |

**bias in measurement of the outcome**

| Signaling question | Response | Supporting information |
| --- | --- | --- |
| 4.1 Was the method of measuring the outcome inappropriate? | N | The method was appropriate. |
| 4.2 Could measurement or ascertainment of the outcome have differed between intervention groups? | N | There was no difference between groups. |
| 4.3 If N/PN/NI to 4.1 and 4.2: Were outcome assessors aware of the intervention received by study participants? | PY | Blinding was not used. |
| 4.4 If Y/PY/NI to 4.3: Could assessment of the outcome have been influenced by knowledge of intervention received? | N | The outcome involves no judgement. |
| 4.5 If Y/PY/NI to 4.4: Is it likely that assessment of the outcome was influenced by knowledge of intervention received? | NA | - |

**bias in selection of the reported result**

| Signaling question | Response | Supporting information |
| --- | --- | --- |
| 5.1 Were the data that produced this result analyzed in accordance with a pre-specified analysis plan that was finalized before unblinded outcome data were available for analysis? | N | Protocol was not available. |
| s the numerical result being assessed likely to have been selected, on the basis of the results, from... |  |  |
| 5.2. ... multiple eligible outcome measurements (e.g. scales, definitions, time points) within the outcome domain? | NI | Protocol was not available. |
| 5.3 ... multiple eligible analyses of the data? | NI | Protocol was not available. |

1. **Wenjun Yang, 2013-2**

**Bias arising from the randomization process**

| Signaling question | Response | Supporting information |
| --- | --- | --- |
| 1.1 Was the allocation sequence random? | NI | The author did not elaborate on what random method was used. |
| 1.2 Was the allocation sequence concealed until participants were enrolled and assigned to interventions? | NI | The author didn't mention whether the allocation sequence was concealed. |
| 1.3 Did baseline differences between intervention groups suggest a problem with the randomization process? | PN | The two groups were comparable. |

**bias due to deviations from the intended interventions (effect of assignment to intervention)**

| Signaling question | Response | Supporting information |
| --- | --- | --- |
| 2.1. Were participants aware of their assigned intervention during the trial? | Y | Since there is no placebo control, it is easy for everyone to know the intervention. |
| 2.2. Were carers and people delivering the interventions aware of participants' assigned intervention during the trial? | Y | Since there is no placebo control, it is easy for everyone to know the intervention. |
| 2.3. If Y/PY/NI to 2.1 or 2.2: Were there deviations from the intended intervention that arose because of the trial context? | NI | No enough information. |
| 2.4 If Y/PY to 2.3: Were these deviations likely to have affected the outcome? | NA | - |
| 2.5. If Y/PY/NI to 2.4: Were these deviations from intended intervention balanced between groups? | NA | - |
| 2.6 Was an appropriate analysis used to estimate the effect of assignment to intervention? | N | ITT analysis was not used. |
| 2.7 If N/PN/NI to 2.6: Was there potential for a substantial impact (on the result) of the failure to analyses participants in the group to which they were randomized? | NI | No enough information. |

**Bias due to missing outcome data**

| Signaling question | Response | Supporting information |
| --- | --- | --- |
| 3.1 Were data for this outcome available for all, or nearly all, participants randomized? | NI | Since we don’t know the number of randomized patients, we cannot tell whether all patients’ data are available. |
| 3.2 If N/PN/NI to 3.1: Is there evidence that the result was not biased by missing outcome data? | PN | No enough information. |
| 3.3 If N/PN to 3.2: Could missingness in the outcome depend on its true value? | NI | No enough information. |
| 3.4 If Y/PY/NI to 3.3: Is it likely that missingness in the outcome depended on its true value? | NI | No enough information. |

**bias in measurement of the outcome**

| Signaling question | Response | Supporting information |
| --- | --- | --- |
| 4.1 Was the method of measuring the outcome inappropriate? | N | The method was appropriate. |
| 4.2 Could measurement or ascertainment of the outcome have differed between intervention groups? | N | There was no difference between groups. |
| 4.3 If N/PN/NI to 4.1 and 4.2: Were outcome assessors aware of the intervention received by study participants? | PY | Blinding was not used. |
| 4.4 If Y/PY/NI to 4.3: Could assessment of the outcome have been influenced by knowledge of intervention received? | N | The outcome involves no judgement. |
| 4.5 If Y/PY/NI to 4.4: Is it likely that assessment of the outcome was influenced by knowledge of intervention received? | NA | - |

**bias in selection of the reported result**

| Signaling question | Response | Supporting information |
| --- | --- | --- |
| 5.1 Were the data that produced this result analyzed in accordance with a pre-specified analysis plan that was finalized before unblinded outcome data were available for analysis? | N | Protocol was not available. |
| s the numerical result being assessed likely to have been selected, on the basis of the results, from... |  |  |
| 5.2. ... multiple eligible outcome measurements (e.g. scales, definitions, time points) within the outcome domain? | NI | Protocol was not available. |
| 5.3 ... multiple eligible analyses of the data? | NI | Protocol was not available. |

**3.Weishan Li, 2012**

**Bias arising from the randomization process**

| Signaling question | Response | Supporting information |
| --- | --- | --- |
| 1.1 Was the allocation sequence random? | NI | The author did not elaborate on what random method was used. |
| 1.2 Was the allocation sequence concealed until participants were enrolled and assigned to interventions? | NI | The author didn't mention whether the allocation sequence was concealed. |
| 1.3 Did baseline differences between intervention groups suggest a problem with the randomization process? | PN | The two groups were comparable. |

**bias due to deviations from the intended interventions (effect of assignment to intervention)**

| Signaling question | Response | Supporting information |
| --- | --- | --- |
| 2.1. Were participants aware of their assigned intervention during the trial? | Y | Since there is no placebo control, it is easy for everyone to know the intervention. |
| 2.2. Were carers and people delivering the interventions aware of participants' assigned intervention during the trial? | Y | Since there is no placebo control, it is easy for everyone to know the intervention. |
| 2.3. If Y/PY/NI to 2.1 or 2.2: Were there deviations from the intended intervention that arose because of the trial context? | NI | No enough information. |
| 2.4 If Y/PY to 2.3: Were these deviations likely to have affected the outcome? | NA | - |
| 2.5. If Y/PY/NI to 2.4: Were these deviations from intended intervention balanced between groups? | NA | - |
| 2.6 Was an appropriate analysis used to estimate the effect of assignment to intervention? | PY | ITT analysis was used. |
| 2.7 If N/PN/NI to 2.6: Was there potential for a substantial impact (on the result) of the failure to analyses participants in the group to which they were randomized? | NA | - |

**Bias due to missing outcome data**

| Signaling question | Response | Supporting information |
| --- | --- | --- |
| 3.1 Were data for this outcome available for all, or nearly all, participants randomized? | NI | Since we don’t know the number of randomized patients, we cannot tell whether all patients’ data are available. |
| 3.2 If N/PN/NI to 3.1: Is there evidence that the result was not biased by missing outcome data? | PN | No enough information. |
| 3.3 If N/PN to 3.2: Could missingness in the outcome depend on its true value? | NI | No enough information. |
| 3.4 If Y/PY/NI to 3.3: Is it likely that missingness in the outcome depended on its true value? | NI | No enough information. |

**bias in measurement of the outcome**

| Signaling question | Response | Supporting information |
| --- | --- | --- |
| 4.1 Was the method of measuring the outcome inappropriate? | N | The method was appropriate. |
| 4.2 Could measurement or ascertainment of the outcome have differed between intervention groups? | N | There was no difference between groups. |
| 4.3 If N/PN/NI to 4.1 and 4.2: Were outcome assessors aware of the intervention received by study participants? | PY | Blinding was not used. |
| 4.4 If Y/PY/NI to 4.3: Could assessment of the outcome have been influenced by knowledge of intervention received? | N | The outcome involves no judgement. |
| 4.5 If Y/PY/NI to 4.4: Is it likely that assessment of the outcome was influenced by knowledge of intervention received? | NA | - |

**bias in selection of the reported result**

| Signaling question | Response | Supporting information |
| --- | --- | --- |
| 5.1 Were the data that produced this result analyzed in accordance with a pre-specified analysis plan that was finalized before unblinded outcome data were available for analysis? | N | Protocol was not available. |
| s the numerical result being assessed likely to have been selected, on the basis of the results, from... |  |  |
| 5.2. ... multiple eligible outcome measurements (e.g. scales, definitions, time points) within the outcome domain? | NI | Protocol was not available. |
| 5.3 ... multiple eligible analyses of the data? | NI | Protocol was not available. |

**4.Yajin Zhang, 2020**

**Bias arising from the randomization process**

| Signaling question | Response | Supporting information |
| --- | --- | --- |
| 1.1 Was the allocation sequence random? | NI | The author did not elaborate on what random method was used. |
| 1.2 Was the allocation sequence concealed until participants were enrolled and assigned to interventions? | NI | The author didn't mention whether the allocation sequence was concealed. |
| 1.3 Did baseline differences between intervention groups suggest a problem with the randomization process? | PN | The two groups were comparable. |

**bias due to deviations from the intended interventions (effect of assignment to intervention)**

| Signaling question | Response | Supporting information |
| --- | --- | --- |
| 2.1. Were participants aware of their assigned intervention during the trial? | Y | Since there is no placebo control, it is easy for everyone to know the intervention. |
| 2.2. Were carers and people delivering the interventions aware of participants' assigned intervention during the trial? | Y | Since there is no placebo control, it is easy for everyone to know the intervention. |
| 2.3. If Y/PY/NI to 2.1 or 2.2: Were there deviations from the intended intervention that arose because of the trial context? | NI | No enough information. |
| 2.4 If Y/PY to 2.3: Were these deviations likely to have affected the outcome? | NA | - |
| 2.5. If Y/PY/NI to 2.4: Were these deviations from intended intervention balanced between groups? | NA | - |
| 2.6 Was an appropriate analysis used to estimate the effect of assignment to intervention? | N | ITT analysis was not used. |
| 2.7 If N/PN/NI to 2.6: Was there potential for a substantial impact (on the result) of the failure to analyses participants in the group to which they were randomized? | NI | No enough information. |

**Bias due to missing outcome data**

| Signaling question | Response | Supporting information |
| --- | --- | --- |
| 3.1 Were data for this outcome available for all, or nearly all, participants randomized? | NI | Since we don’t know the number of randomized patients, we cannot tell whether all patients’ data are available. |
| 3.2 If N/PN/NI to 3.1: Is there evidence that the result was not biased by missing outcome data? | PN | No enough information. |
| 3.3 If N/PN to 3.2: Could missingness in the outcome depend on its true value? | NI | No enough information. |
| 3.4 If Y/PY/NI to 3.3: Is it likely that missingness in the outcome depended on its true value? | NI | No enough information. |

**bias in measurement of the outcome**

| Signaling question | Response | Supporting information |
| --- | --- | --- |
| 4.1 Was the method of measuring the outcome inappropriate? | N | The method was appropriate. |
| 4.2 Could measurement or ascertainment of the outcome have differed between intervention groups? | N | There was no difference between groups. |
| 4.3 If N/PN/NI to 4.1 and 4.2: Were outcome assessors aware of the intervention received by study participants? | PY | Blinding was not used. |
| 4.4 If Y/PY/NI to 4.3: Could assessment of the outcome have been influenced by knowledge of intervention received? | N | The outcome involves no judgement. |
| 4.5 If Y/PY/NI to 4.4: Is it likely that assessment of the outcome was influenced by knowledge of intervention received? | NA | - |

**bias in selection of the reported result**

| Signaling question | Response | Supporting information |
| --- | --- | --- |
| 5.1 Were the data that produced this result analyzed in accordance with a pre-specified analysis plan that was finalized before unblinded outcome data were available for analysis? | N | Protocol was not available. |
| s the numerical result being assessed likely to have been selected, on the basis of the results, from... |  |  |
| 5.2. ... multiple eligible outcome measurements (e.g. scales, definitions, time points) within the outcome domain? | NI | Protocol was not available. |
| 5.3 ... multiple eligible analyses of the data? | NI | Protocol was not available. |

**5.Qiang Guo, 2018**

**Bias arising from the randomization process**

| Signaling question | Response | Supporting information |
| --- | --- | --- |
| 1.1 Was the allocation sequence random? | PN | The author did not report on what random method was used. |
| 1.2 Was the allocation sequence concealed until participants were enrolled and assigned to interventions? | PN | The author didn't mention whether the allocation sequence was concealed. |
| 1.3 Did baseline differences between intervention groups suggest a problem with the randomization process? | PN | The two groups were comparable. |

**bias due to deviations from the intended interventions (effect of assignment to intervention)**

| Signaling question | Response | Supporting information |
| --- | --- | --- |
| 2.1. Were participants aware of their assigned intervention during the trial? | Y | Since there is no placebo control, it is easy for everyone to know the intervention. |
| 2.2. Were carers and people delivering the interventions aware of participants' assigned intervention during the trial? | Y | Since there is no placebo control, it is easy for everyone to know the intervention. |
| 2.3. If Y/PY/NI to 2.1 or 2.2: Were there deviations from the intended intervention that arose because of the trial context? | NI | No enough information. |
| 2.4 If Y/PY to 2.3: Were these deviations likely to have affected the outcome? | NA | - |
| 2.5. If Y/PY/NI to 2.4: Were these deviations from intended intervention balanced between groups? | NA | - |
| 2.6 Was an appropriate analysis used to estimate the effect of assignment to intervention? | N | ITT analysis was not used. |
| 2.7 If N/PN/NI to 2.6: Was there potential for a substantial impact (on the result) of the failure to analyses participants in the group to which they were randomized? | NI | No enough information. |

**Bias due to missing outcome data**

| Signaling question | Response | Supporting information |
| --- | --- | --- |
| 3.1 Were data for this outcome available for all, or nearly all, participants randomized? | NI | Since we don’t know the number of randomized patients, we cannot tell whether all patients’ data are available. |
| 3.2 If N/PN/NI to 3.1: Is there evidence that the result was not biased by missing outcome data? | PN | No enough information. |
| 3.3 If N/PN to 3.2: Could missingness in the outcome depend on its true value? | NI | No enough information. |
| 3.4 If Y/PY/NI to 3.3: Is it likely that missingness in the outcome depended on its true value? | NI | No enough information. |

**bias in measurement of the outcome**

| Signaling question | Response | Supporting information |
| --- | --- | --- |
| 4.1 Was the method of measuring the outcome inappropriate? | N | The method was appropriate. |
| 4.2 Could measurement or ascertainment of the outcome have differed between intervention groups? | N | There was no difference between groups. |
| 4.3 If N/PN/NI to 4.1 and 4.2: Were outcome assessors aware of the intervention received by study participants? | PY | Blinding was not used. |
| 4.4 If Y/PY/NI to 4.3: Could assessment of the outcome have been influenced by knowledge of intervention received? | N | The outcome involves no judgement. |
| 4.5 If Y/PY/NI to 4.4: Is it likely that assessment of the outcome was influenced by knowledge of intervention received? | NA | - |

**bias in selection of the reported result**

| Signaling question | Response | Supporting information |
| --- | --- | --- |
| 5.1 Were the data that produced this result analyzed in accordance with a pre-specified analysis plan that was finalized before unblinded outcome data were available for analysis? | N | Protocol was not available. |
| s the numerical result being assessed likely to have been selected, on the basis of the results, from... |  |  |
| 5.2. ... multiple eligible outcome measurements (e.g. scales, definitions, time points) within the outcome domain? | NI | Protocol was not available. |
| 5.3 ... multiple eligible analyses of the data? | NI | Protocol was not available. |

**6.Xiang Ding, 2018**

**Bias arising from the randomization process**

| Signaling question | Response | Supporting information |
| --- | --- | --- |
| 1.1 Was the allocation sequence random? | PN | The author used wrong random method. |
| 1.2 Was the allocation sequence concealed until participants were enrolled and assigned to interventions? | PN | The author didn't mention whether the allocation sequence was concealed. |
| 1.3 Did baseline differences between intervention groups suggest a problem with the randomization process? | Y | There are some important baseline characteristics that have not been reported. |

**bias due to deviations from the intended interventions (effect of assignment to intervention)**

| Signaling question | Response | Supporting information |
| --- | --- | --- |
| 2.1. Were participants aware of their assigned intervention during the trial? | Y | Since there is no placebo control, it is easy for everyone to know the intervention. |
| 2.2. Were carers and people delivering the interventions aware of participants' assigned intervention during the trial? | Y | Since there is no placebo control, it is easy for everyone to know the intervention. |
| 2.3. If Y/PY/NI to 2.1 or 2.2: Were there deviations from the intended intervention that arose because of the trial context? | NI | No enough information. |
| 2.4 If Y/PY to 2.3: Were these deviations likely to have affected the outcome? | NA | - |
| 2.5. If Y/PY/NI to 2.4: Were these deviations from intended intervention balanced between groups? | NA | - |
| 2.6 Was an appropriate analysis used to estimate the effect of assignment to intervention? | N | ITT analysis was not used. |
| 2.7 If N/PN/NI to 2.6: Was there potential for a substantial impact (on the result) of the failure to analyses participants in the group to which they were randomized? | NI | No enough information. |

**Bias due to missing outcome data**

| Signaling question | Response | Supporting information |
| --- | --- | --- |
| 3.1 Were data for this outcome available for all, or nearly all, participants randomized? | NI | Since we don’t know the number of randomized patients, we cannot tell whether all patients’ data are available. |
| 3.2 If N/PN/NI to 3.1: Is there evidence that the result was not biased by missing outcome data? | PN | No enough information. |
| 3.3 If N/PN to 3.2: Could missingness in the outcome depend on its true value? | NI | No enough information. |
| 3.4 If Y/PY/NI to 3.3: Is it likely that missingness in the outcome depended on its true value? | NI | No enough information. |

**bias in measurement of the outcome**

| Signaling question | Response | Supporting information |
| --- | --- | --- |
| 4.1 Was the method of measuring the outcome inappropriate? | N | The method was appropriate. |
| 4.2 Could measurement or ascertainment of the outcome have differed between intervention groups? | N | There was no difference between groups. |
| 4.3 If N/PN/NI to 4.1 and 4.2: Were outcome assessors aware of the intervention received by study participants? | PY | Blinding was not used. |
| 4.4 If Y/PY/NI to 4.3: Could assessment of the outcome have been influenced by knowledge of intervention received? | N | The outcome involves no judgement. |
| 4.5 If Y/PY/NI to 4.4: Is it likely that assessment of the outcome was influenced by knowledge of intervention received? | NA | - |

**bias in selection of the reported result**

| Signaling question | Response | Supporting information |
| --- | --- | --- |
| 5.1 Were the data that produced this result analyzed in accordance with a pre-specified analysis plan that was finalized before unblinded outcome data were available for analysis? | N | Protocol was not available. |
| s the numerical result being assessed likely to have been selected, on the basis of the results, from... |  |  |
| 5.2. ... multiple eligible outcome measurements (e.g. scales, definitions, time points) within the outcome domain? | NI | Protocol was not available. |
| 5.3 ... multiple eligible analyses of the data? | NI | Protocol was not available. |

**7.Jiajun Feng, 2019**

**Bias arising from the randomization process**

| Signaling question | Response | Supporting information |
| --- | --- | --- |
| 1.1 Was the allocation sequence random? | Y | Random number table. |
| 1.2 Was the allocation sequence concealed until participants were enrolled and assigned to interventions? | NI | The author didn't mention whether the allocation sequence was concealed. |
| 1.3 Did baseline differences between intervention groups suggest a problem with the randomization process? | PN | The participants’ baseline data seems to be balanced. |

**bias due to deviations from the intended interventions (effect of assignment to intervention)**

| Signaling question | Response | Supporting information |
| --- | --- | --- |
| 2.1. Were participants aware of their assigned intervention during the trial? | Y | Since there is no placebo control, it is easy for everyone to know the intervention. |
| 2.2. Were carers and people delivering the interventions aware of participants' assigned intervention during the trial? | Y | Since there is no placebo control, it is easy for everyone to know the intervention. |
| 2.3. If Y/PY/NI to 2.1 or 2.2: Were there deviations from the intended intervention that arose because of the trial context? | NI | No enough information. |
| 2.4 If Y/PY to 2.3: Were these deviations likely to have affected the outcome? | NA | - |
| 2.5. If Y/PY/NI to 2.4: Were these deviations from intended intervention balanced between groups? | NA | - |
| 2.6 Was an appropriate analysis used to estimate the effect of assignment to intervention? | N | ITT analysis was not used. |
| 2.7 If N/PN/NI to 2.6: Was there potential for a substantial impact (on the result) of the failure to analyses participants in the group to which they were randomized? | NI | No enough information. |

**Bias due to missing outcome data**

| Signaling question | Response | Supporting information |
| --- | --- | --- |
| 3.1 Were data for this outcome available for all, or nearly all, participants randomized? | NI | Since we don’t know the number of randomized patients, we cannot tell whether all patients’ data are available. |
| 3.2 If N/PN/NI to 3.1: Is there evidence that the result was not biased by missing outcome data? | PN | No enough information. |
| 3.3 If N/PN to 3.2: Could missingness in the outcome depend on its true value? | NI | No enough information. |
| 3.4 If Y/PY/NI to 3.3: Is it likely that missingness in the outcome depended on its true value? | NI | No enough information. |

**bias in measurement of the outcome**

| Signaling question | Response | Supporting information |
| --- | --- | --- |
| 4.1 Was the method of measuring the outcome inappropriate? | N | The method was appropriate. |
| 4.2 Could measurement or ascertainment of the outcome have differed between intervention groups? | N | There was no difference between groups. |
| 4.3 If N/PN/NI to 4.1 and 4.2: Were outcome assessors aware of the intervention received by study participants? | PY | Blinding was not used. |
| 4.4 If Y/PY/NI to 4.3: Could assessment of the outcome have been influenced by knowledge of intervention received? | N | The outcome involves no judgement. |
| 4.5 If Y/PY/NI to 4.4: Is it likely that assessment of the outcome was influenced by knowledge of intervention received? | NA | - |

**bias in selection of the reported result**

| Signaling question | Response | Supporting information |
| --- | --- | --- |
| 5.1 Were the data that produced this result analyzed in accordance with a pre-specified analysis plan that was finalized before unblinded outcome data were available for analysis? | N | Protocol was not available. |
| s the numerical result being assessed likely to have been selected, on the basis of the results, from... |  |  |
| 5.2. ... multiple eligible outcome measurements (e.g. scales, definitions, time points) within the outcome domain? | NI | Protocol was not available. |
| 5.3 ... multiple eligible analyses of the data? | NI | Protocol was not available. |

**8.Wen Cheng, 2018**

**Bias arising from the randomization process**

| Signaling question | Response | Supporting information |
| --- | --- | --- |
| 1.1 Was the allocation sequence random? | NI | The author did not report how the allocation sequence was generated. |
| 1.2 Was the allocation sequence concealed until participants were enrolled and assigned to interventions? | NI | The author didn't mention whether the allocation sequence was concealed. |
| 1.3 Did baseline differences between intervention groups suggest a problem with the randomization process? | PN | The participants’ baseline data seems to be balanced. |

**bias due to deviations from the intended interventions (effect of assignment to intervention)**

| Signaling question | Response | Supporting information |
| --- | --- | --- |
| 2.1. Were participants aware of their assigned intervention during the trial? | Y | Since there is no placebo control, it is easy for everyone to know the intervention. |
| 2.2. Were carers and people delivering the interventions aware of participants' assigned intervention during the trial? | Y | Since there is no placebo control, it is easy for everyone to know the intervention. |
| 2.3. If Y/PY/NI to 2.1 or 2.2: Were there deviations from the intended intervention that arose because of the trial context? | NI | No enough information. |
| 2.4 If Y/PY to 2.3: Were these deviations likely to have affected the outcome? | NA | - |
| 2.5. If Y/PY/NI to 2.4: Were these deviations from intended intervention balanced between groups? | NA | - |
| 2.6 Was an appropriate analysis used to estimate the effect of assignment to intervention? | N | ITT analysis was not used. |
| 2.7 If N/PN/NI to 2.6: Was there potential for a substantial impact (on the result) of the failure to analyses participants in the group to which they were randomized? | NI | No enough information. |

**Bias due to missing outcome data**

| Signaling question | Response | Supporting information |
| --- | --- | --- |
| 3.1 Were data for this outcome available for all, or nearly all, participants randomized? | NI | Since we don’t know the number of randomized patients, we cannot tell whether all patients’ data are available. |
| 3.2 If N/PN/NI to 3.1: Is there evidence that the result was not biased by missing outcome data? | PN | No enough information. |
| 3.3 If N/PN to 3.2: Could missingness in the outcome depend on its true value? | NI | No enough information. |
| 3.4 If Y/PY/NI to 3.3: Is it likely that missingness in the outcome depended on its true value? | NI | No enough information. |

**bias in measurement of the outcome**

| Signaling question | Response | Supporting information |
| --- | --- | --- |
| 4.1 Was the method of measuring the outcome inappropriate? | N | The method was appropriate. |
| 4.2 Could measurement or ascertainment of the outcome have differed between intervention groups? | N | There was no difference between groups. |
| 4.3 If N/PN/NI to 4.1 and 4.2: Were outcome assessors aware of the intervention received by study participants? | PY | Blinding was not used. |
| 4.4 If Y/PY/NI to 4.3: Could assessment of the outcome have been influenced by knowledge of intervention received? | N | The outcome involves no judgement. |
| 4.5 If Y/PY/NI to 4.4: Is it likely that assessment of the outcome was influenced by knowledge of intervention received? | NA | - |

**bias in selection of the reported result**

| Signaling question | Response | Supporting information |
| --- | --- | --- |
| 5.1 Were the data that produced this result analyzed in accordance with a pre-specified analysis plan that was finalized before unblinded outcome data were available for analysis? | N | Protocol was not available. |
| s the numerical result being assessed likely to have been selected, on the basis of the results, from... |  |  |
| 5.2. ... multiple eligible outcome measurements (e.g. scales, definitions, time points) within the outcome domain? | NI | Protocol was not available. |
| 5.3 ... multiple eligible analyses of the data? | NI | Protocol was not available. |

**9.Jin Lin, 2015**

**Bias arising from the randomization process**

| Signaling question | Response | Supporting information |
| --- | --- | --- |
| 1.1 Was the allocation sequence random? | PN | The author did not report how the allocation sequence was generated. |
| 1.2 Was the allocation sequence concealed until participants were enrolled and assigned to interventions? | PN | The author didn't mention whether the allocation sequence was concealed. |
| 1.3 Did baseline differences between intervention groups suggest a problem with the randomization process? | PN | The participants’ baseline data seems to be balanced. |

**bias due to deviations from the intended interventions (effect of assignment to intervention)**

| Signaling question | Response | Supporting information |
| --- | --- | --- |
| 2.1. Were participants aware of their assigned intervention during the trial? | Y | Since there is no placebo control, it is easy for everyone to know the intervention. |
| 2.2. Were carers and people delivering the interventions aware of participants' assigned intervention during the trial? | Y | Since there is no placebo control, it is easy for everyone to know the intervention. |
| 2.3. If Y/PY/NI to 2.1 or 2.2: Were there deviations from the intended intervention that arose because of the trial context? | NI | No enough information. |
| 2.4 If Y/PY to 2.3: Were these deviations likely to have affected the outcome? | NA | - |
| 2.5. If Y/PY/NI to 2.4: Were these deviations from intended intervention balanced between groups? | NA | - |
| 2.6 Was an appropriate analysis used to estimate the effect of assignment to intervention? | N | ITT analysis was not used. |
| 2.7 If N/PN/NI to 2.6: Was there potential for a substantial impact (on the result) of the failure to analyses participants in the group to which they were randomized? | NI | No enough information. |

**Bias due to missing outcome data**

| Signaling question | Response | Supporting information |
| --- | --- | --- |
| 3.1 Were data for this outcome available for all, or nearly all, participants randomized? | NI | Since we don’t know the number of randomized patients, we cannot tell whether all patients’ data are available. |
| 3.2 If N/PN/NI to 3.1: Is there evidence that the result was not biased by missing outcome data? | PN | No enough information. |
| 3.3 If N/PN to 3.2: Could missingness in the outcome depend on its true value? | NI | No enough information. |
| 3.4 If Y/PY/NI to 3.3: Is it likely that missingness in the outcome depended on its true value? | NI | No enough information. |

**bias in measurement of the outcome**

| Signaling question | Response | Supporting information |
| --- | --- | --- |
| 4.1 Was the method of measuring the outcome inappropriate? | N | The method was appropriate. |
| 4.2 Could measurement or ascertainment of the outcome have differed between intervention groups? | N | There was no difference between groups. |
| 4.3 If N/PN/NI to 4.1 and 4.2: Were outcome assessors aware of the intervention received by study participants? | PY | Blinding was not used. |
| 4.4 If Y/PY/NI to 4.3: Could assessment of the outcome have been influenced by knowledge of intervention received? | N | The outcome involves no judgement. |
| 4.5 If Y/PY/NI to 4.4: Is it likely that assessment of the outcome was influenced by knowledge of intervention received? | NA | - |

**bias in selection of the reported result**

| Signaling question | Response | Supporting information |
| --- | --- | --- |
| 5.1 Were the data that produced this result analyzed in accordance with a pre-specified analysis plan that was finalized before unblinded outcome data were available for analysis? | N | Protocol was not available. |
| s the numerical result being assessed likely to have been selected, on the basis of the results, from... |  |  |
| 5.2. ... multiple eligible outcome measurements (e.g. scales, definitions, time points) within the outcome domain? | NI | Protocol was not available. |
| 5.3 ... multiple eligible analyses of the data? | NI | Protocol was not available. |
