# Supplementary material for: Effect of Huang-Lian Jie-Du Decoction on Glucose and Lipid Metabolism in Type 2 Diabetes Mellitus: A Systematic Review and Meta-Analysis
Source: Front Pharmacol. 2021 Apr 29;12:648861. doi: 10.3389/fphar.2021.648861 (PMC8117159; doi:10.3389/fphar.2021.648861)
Supplement: Supplementary file 11 [file DataSheet2.docx]

**A list of excluded studies by reading full text.**

**Study design:**

1. Gao K, Yang R, Zhang J, et al. Effects of Qijian mixture on type 2 diabetes assessed by metabonomics, gut microbiota and network pharmacology. Pharmacological research. 2018;130:93-109.
2. Qian Guangfang. Huanglian Jiedu Decoction in the treatment of type 2 diabetes [J]. Chinese practical medicine. 2012; 7 (14): 234-235.

**Intervention:**

1. Chen Mingzhi. Clinical effect of Huanglian Jiedu Decoction in the adjuvant treatment of diabetes [J]. Seeking medical advice (second half of the month). 2013; 11 (08): 36.
2. Chen yingluan. Huanglian Jiedu decoction combined with western medicine in the treatment of type 2 diabetes with Yin deficiency and heat excess syndrome and its influence on blood glucose level [J]. Clinical Journal of Chinese medicine. 2019; 11 (17): 50-52.
3. Han Ying. Effect of Huanglian Jiedu Decoction on blood glucose level in patients with type 2 diabetes with Yin deficiency and heat excess syndrome [J]. Diabetes world. 2020; 17 (3): 61.
4. He bin. Effect of Coptis chinensis related Chinese herbal compound on diabetes and its complications [J]. Taiwan pharmaceutical. 2013; 25 (09): 139-140.
5. Ji Wenjuan. Clinical study of Huanglian Jiedu decoction combined with basic insulin in the treatment of obese newly diagnosed type 2 diabetes mellitus [Master], Shandong University of traditional Chinese medicine; 2013.
6. Ji Wenjuan, Yang Wenjun. Clinical study of Huanglian Jiedu decoction combined with basic insulin in the treatment of 30 cases of obese newly diagnosed type 2 diabetes [J]. Journal of Gansu University of traditional Chinese medicine. 2013; 30 (5): 28-30.
7. Li Li. Clinical intervention study of Huanglian Jiedu Decoction on newly obese patients with type 2 diabetes [Master], Shandong University of traditional Chinese medicine; 2013.
8. Li Song, Han Jinghui, Meng Changhai. Effects of Huanglian Jiedu decoction combined with Compound Danshen Dripping Pills on islet β cell function and serum CRP and ICAM-1 levels in newly diagnosed type 2 diabetes mellitus [J]. Acta Sinica Sinica. 2018; 33 (04): 560-564.
9. Li Xin. Clinical efficacy of Huanglian Jiedu Decoction in the treatment of type 2 diabetes mellitus complicated with coronary heart disease [J]. Healthy friend. 2019 (2): 34.
10. Liu Qiang. Huanglian Jiedu decoction combined with insulin in the treatment of diabetes mellitus and complications: a randomized parallel controlled study [J]. Journal of practical Chinese medicine internal medicine. 2013; 27 (12): 91-92.
11. Lu Chunru. Clinical observation of Huanglian Jiedu Decoction in the treatment of type 2 diabetes mellitus complicated with coronary heart disease [J]. Diabetes new world. 2019; 22 (19): 74-75.
12. Niu Tao. Huanglian Jiedu mixture in the treatment of 32 cases of obese type 2 diabetes [J]. Shandong medicine. 2011; 51 (37).
13. Tan Juan, Zhang Geping. Effect observation of Huanglian Jiedu Decoction on 30 cases of obese type 2 diabetes mellitus. 2016.
14. Tian Wenhong. Huanglian Jiedu Decoction and Zengye Decoction in the treatment of 32 cases of diabetes [J]. Shaanxi traditional Chinese medicine. 2008; 0 (12).
15. Tu Chunlian. Clinical study of Huanglian Jiedu Decoction on obese type 2 diabetes mellitus [J]. Acta Sinica Sinica. 2015; 30 (5).
16. Tu Chunlian, Li Hongsheng. Clinical study of Huanglian Jiedu Decoction on obese type 2 diabetes mellitus [J]. Acta Sinica Sinica. 2015; 30 (05): 644-646.
17. Wen Zhanjun. Clinical effect of Huanglian Jiedu Decoction in the treatment of diabetes [J]. China Health Care & nutrition. 2017; 27 (6): 317-318.
18. Xiang Fangyu. Huanglian Jiedu decoction combined with western medicine in the treatment of newly diagnosed type 2 diabetes mellitus due to heat Sheng Shangjin [J]. Everyone's health (middle Edition). For all health. 2018; 12 (2): 168.
19. Yao Wei. Huanglian Jiedu decoction combined with Shenqi Jiangtang granule in the treatment of 74 newly diagnosed type 2 diabetes mellitus [J]. Modern diagnosis and treatment. 2018; 29 (10): 1537-1538.
20. Yin Nan, Wei Zheng, Wang Xiaoou. Clinical effect of Huanglian Jiedu decoction combined with abdominal acupuncture on obese type 2 diabetes mellitus [J]. Diabetes new world. 2020; 23 (03): 1-2,
21. Zhang Lei, Yang Yiping, Chen Xinghua. Clinical effect of Huanglian Jiedu Decoction on diabetic patients with perianal abscess surgery and the influence of inflammatory factors [J]. Chinese Journal of health inspection. 2018; 28 (05): 565-567.
22. Zhang Lili. Huanglian Jiedu decoction combined with western medicine in the treatment of newly diagnosed type 2 diabetes mellitus due to heat Sheng Shangjin [J]. Journal of practical Chinese medicine internal medicine. 2017; 31 (11): 34-37.
23. Zhang Meng. Intervention effect of heat clearing and detoxification therapy on inflammatory factors in type 2 diabetes mellitus [Master], Shandong University of traditional Chinese medicine; 2006.
24. Zhang Xitao. Clinical observation of Huanglian Jiedu decoction combined with metformin in the treatment of type 2 diabetes [J]. Jilin Medical. 2014; 35 (16): 3471-3473.
25. Zheng bin. Clinical observation of Huanglian Jiedu decoction combined with metformin in the treatment of type 2 diabetes [J]. Inner Mongolia traditional Chinese medicine. 2016; 35 (01): 67-68.

**Participant:**

1. He Xiaolian. Application of Huanglian Jiedu Decoction in prevention and treatment of cardiovascular diseases [J]. Chinese PLA Journal of preventive medicine. 2019; 37 (6).
2. Hu Lu Ming. The relationship between blood glucose changes and MODS and the effect of Huanglian Jiedu Decoction on them [Master], Guangzhou University of traditional Chinese medicine; 2017.
3. Zhang Hongmin, Yang Mei. Effect of Huanglian Jiedu Decoction on inflammatory factors and protection of intestinal barrier in patients with severe diabetic foot infection [J]. Hebei Traditional Chinese medicine. 2019; 41 (08): 1152-1156.

**Awaiting classification:**

1. Li Li. To investigate the effect of Huanglian Jiedu Decoction on islet function in newly obese patients with type 2 diabetes mellitus. Paper presented at: the 19th national conference on diabetes mellitus of Chinese Medicine Association Diabetes Branch, Chinese Association of Chinese medicine; Hefei, Anhui, China.
2. Li Weishan. Clinical observation of Huanglian Jiedu Decoction in the treatment of diabetes mellitus complicated with carotid atherosclerosis. Paper presented at: the 19th Chinese Medicine Diabetes Conference of diabetes branch, Chinese society of traditional Chinese medicine; Hefei, Anhui, China.
