# Supplementary material for: Effect of Huang-Lian Jie-Du Decoction on Glucose and Lipid Metabolism in Type 2 Diabetes Mellitus: A Systematic Review and Meta-Analysis
Source: Front Pharmacol. 2021 Apr 29;12:648861. doi: 10.3389/fphar.2021.648861 (PMC8117159; doi:10.3389/fphar.2021.648861)
Supplement: Supplementary file 15 [file DataSheet1.docx]

Supplementary material 1: Search strategies for databases.

**PubMed**

| Search | Add to builder | Query | Iterms found | Time |
| --- | --- | --- | --- | --- |
| #5 | Add | ("diabetes mellitus, type 2"[MeSH Terms] OR (((((((((((((((((((((((((((((("diabetes mellitus noninsulin dependent"[Title/Abstract] OR ((("diabetes mellitus"[MeSH Terms] OR ("Diabetes"[All Fields] AND "Mellitus"[All Fields])) OR "diabetes mellitus"[All Fields]) AND "Ketosis-Resistant"[Title/Abstract])) OR ((("diabetes mellitus"[MeSH Terms] OR ("Diabetes"[All Fields] AND "Mellitus"[All Fields])) OR "diabetes mellitus"[All Fields]) AND "Ketosis-Resistant"[Title/Abstract])) OR "ketosis resistant diabetes mellitus"[Title/Abstract]) OR "diabetes mellitus non insulin dependent"[Title/Abstract]) OR "diabetes mellitus non insulin dependent"[Title/Abstract]) OR "non insulin dependent diabetes mellitus"[Title/Abstract]) OR "diabetes mellitus stable"[Title/Abstract]) OR "stable diabetes mellitus"[Title/Abstract]) OR "diabetes mellitus type ii"[Title/Abstract]) OR "NIDDM"[Title/Abstract]) OR "diabetes mellitus noninsulin dependent"[Title/Abstract]) OR "diabetes mellitus maturity onset"[Title/Abstract]) OR "diabetes mellitus maturity onset"[Title/Abstract]) OR "maturity onset diabetes mellitus"[Title/Abstract]) OR "maturity onset diabetes mellitus"[Title/Abstract]) OR "MODY"[Title/Abstract]) OR "diabetes mellitus slow onset"[Title/Abstract]) OR "diabetes mellitus slow onset"[Title/Abstract]) OR ("Slow-Onset"[All Fields] AND "diabetes mellitus"[Title/Abstract])) OR "type 2 diabetes mellitus"[Title/Abstract]) OR "noninsulin dependent diabetes mellitus"[Title/Abstract]) OR "noninsulin dependent diabetes mellitus"[Title/Abstract]) OR "maturity onset diabetes"[Title/Abstract]) OR "diabetes maturity onset"[Title/Abstract]) OR "maturity onset diabetes"[Title/Abstract]) OR "type 2 diabetes"[Title/Abstract]) OR "diabetes type 2"[Title/Abstract]) OR "diabetes mellitus adult onset"[Title/Abstract]) OR "adult onset diabetes mellitus"[Title/Abstract]) OR "diabetes mellitus adult onset"[Title/Abstract])) AND ((((((((("coptis"[MeSH Terms] OR "coptis"[All Fields]) AND "Decoction"[Title/Abstract]) OR (("coptis"[MeSH Terms] OR "coptis"[All Fields]) AND "Tang"[Title/Abstract])) OR (("coptis"[MeSH Terms] OR "coptis"[All Fields]) AND "jiedu decoction"[Title/Abstract])) OR (("coptis"[MeSH Terms] OR "coptis"[All Fields]) AND "jiedu tang"[Title/Abstract])) OR (("coptis"[MeSH Terms] OR "coptis"[All Fields]) AND "Jiedu"[Title/Abstract])) OR "huanglian jiedu decoction"[Title/Abstract]) OR "huanglian jiedu tang"[Title/Abstract]) OR "huanglian jiedu"[Title/Abstract]) | 10 | 23:42:01 |
| #4 | Add | Search: (((((((Coptis Toxin-Resoiving Decoction[Title/Abstract]) OR (Coptis Toxin-Resoiving Tang[Title/Abstract])) OR (Coptis Jiedu Decoction[Title/Abstract])) OR (Coptis Jiedu tang[Title/Abstract])) OR (Coptis Jiedu[Title/Abstract])) OR (Huanglian Jiedu Decoction[Title/Abstract])) OR (Huanglian Jiedu tang[Title/Abstract])) OR (Huanglian Jiedu[Title/Abstract]) | 140 | 23：411：17 |
| #3 | Add | Search: #1 OR #2 | 184394 | 23:33:03 |
| #2 | Add | Search: ((((((((((((((((((((((((((((((Diabetes Mellitus, Noninsulin-Dependent[Title/Abstract]) OR (Diabetes Mellitus, Ketosis-Resistant[Title/Abstract])) OR (Diabetes Mellitus, Ketosis Resistant[Title/Abstract])) OR (Ketosis-Resistant Diabetes Mellitus[Title/Abstract])) OR (Diabetes Mellitus, Non Insulin Dependent[Title/Abstract])) OR (Diabetes Mellitus, Non-Insulin-Dependent[Title/Abstract])) OR (Non-Insulin-Dependent Diabetes Mellitus[Title/Abstract])) OR (Diabetes Mellitus, Stable[Title/Abstract])) OR (Stable Diabetes Mellitus[Title/Abstract])) OR (Diabetes Mellitus, Type II[Title/Abstract])) OR (NIDDM[Title/Abstract])) OR (Diabetes Mellitus, Noninsulin Dependent[Title/Abstract])) OR (Diabetes Mellitus, Maturity-Onset[Title/Abstract])) OR (Diabetes Mellitus, Maturity Onset[Title/Abstract])) OR (Maturity-Onset Diabetes Mellitus[Title/Abstract])) OR (Maturity Onset Diabetes Mellitus[Title/Abstract])) OR (MODY[Title/Abstract])) OR (Diabetes Mellitus, Slow-Onset[Title/Abstract])) OR (Diabetes Mellitus, Slow Onset[Title/Abstract])) OR (Slow-Onset Diabetes Mellitus[Title/Abstract])) OR (Type 2 Diabetes Mellitus[Title/Abstract])) OR (Noninsulin-Dependent Diabetes Mellitus[Title/Abstract])) OR (Noninsulin Dependent Diabetes Mellitus[Title/Abstract])) OR (Maturity-Onset Diabetes[Title/Abstract])) OR (Diabetes, Maturity-Onset[Title/Abstract])) OR (Maturity Onset Diabetes[Title/Abstract])) OR (Type 2 Diabetes[Title/Abstract])) OR (Diabetes, Type 2[Title/Abstract])) OR (Diabetes Mellitus, Adult-Onset[Title/Abstract])) OR (Adult-Onset Diabetes Mellitus[Title/Abstract])) OR (Diabetes Mellitus, Adult Onset[Title/Abstract]) | 139017 | 23:31:17 |
| #1 | Add | Search: "Diabetes Mellitus, Type 2"[Mesh] | 132389 | 23:26:00 |

**
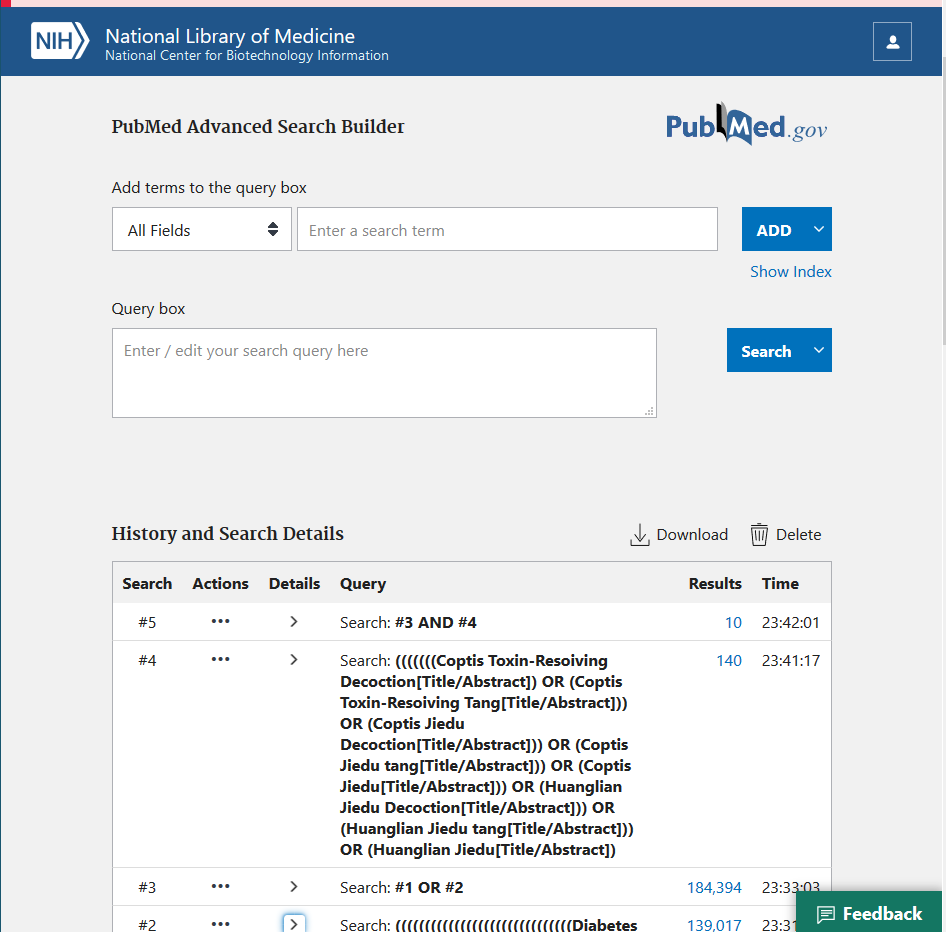
**

**Embase**

**
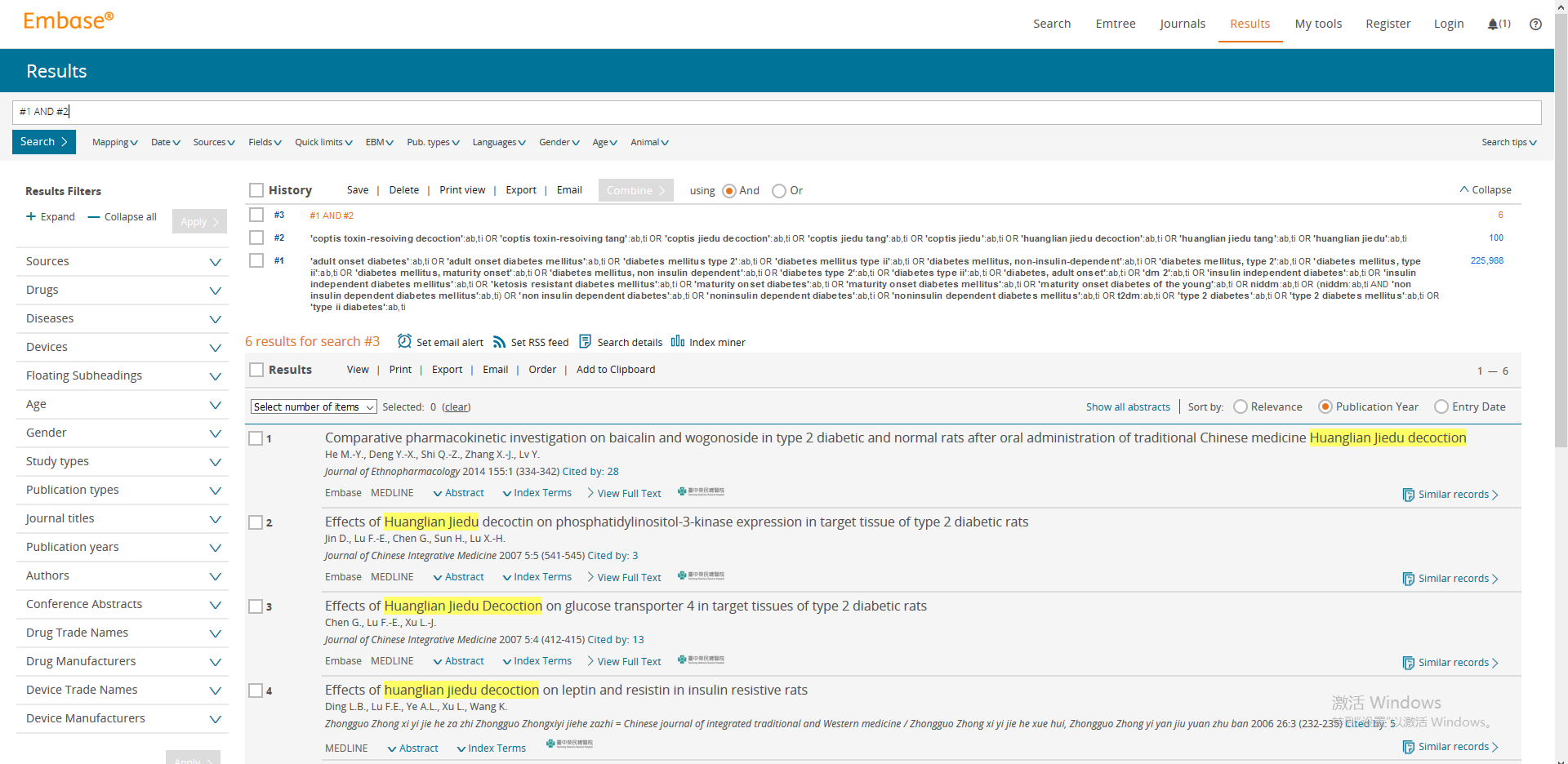
**

**CENTRAL**

**
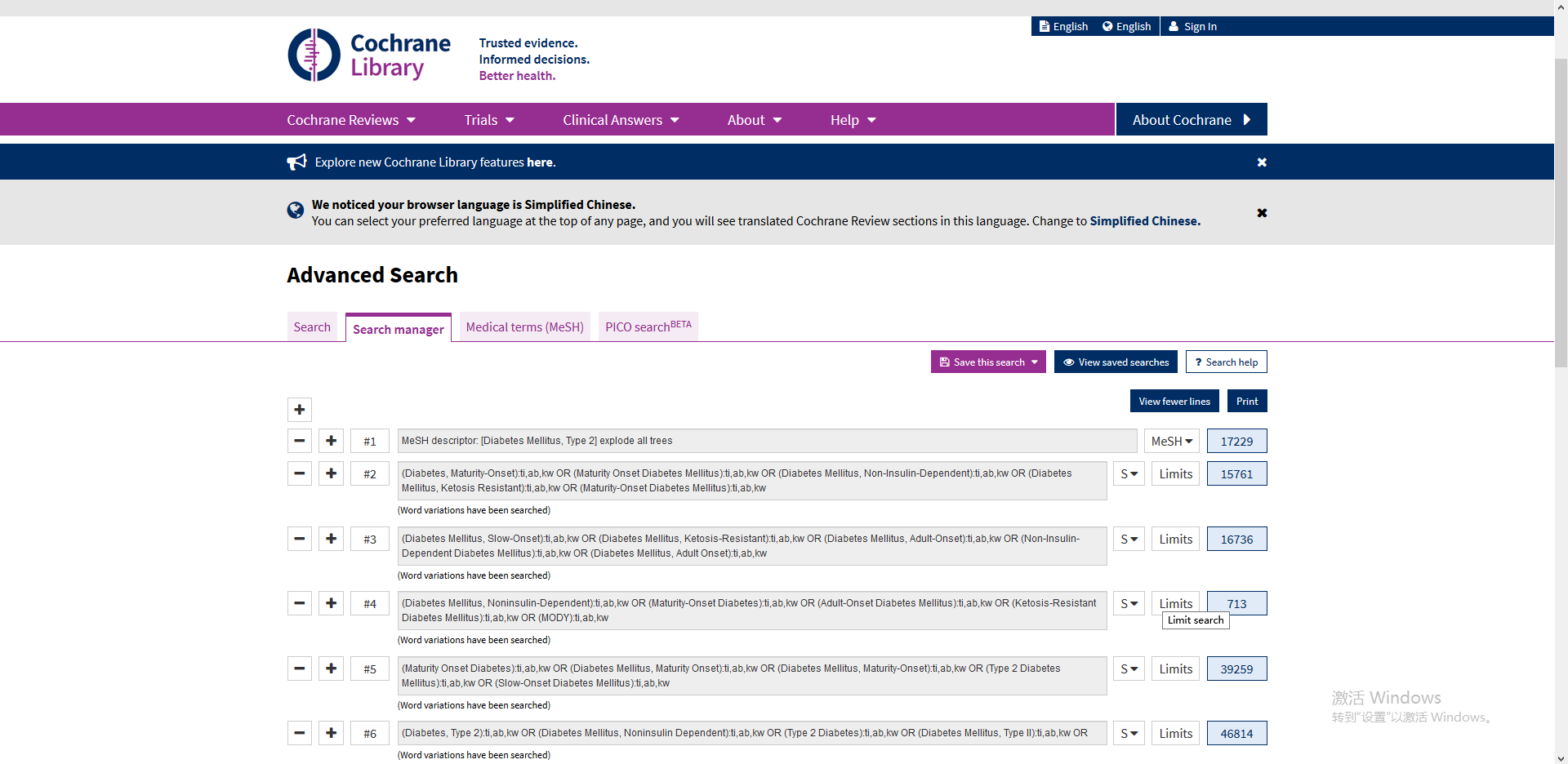

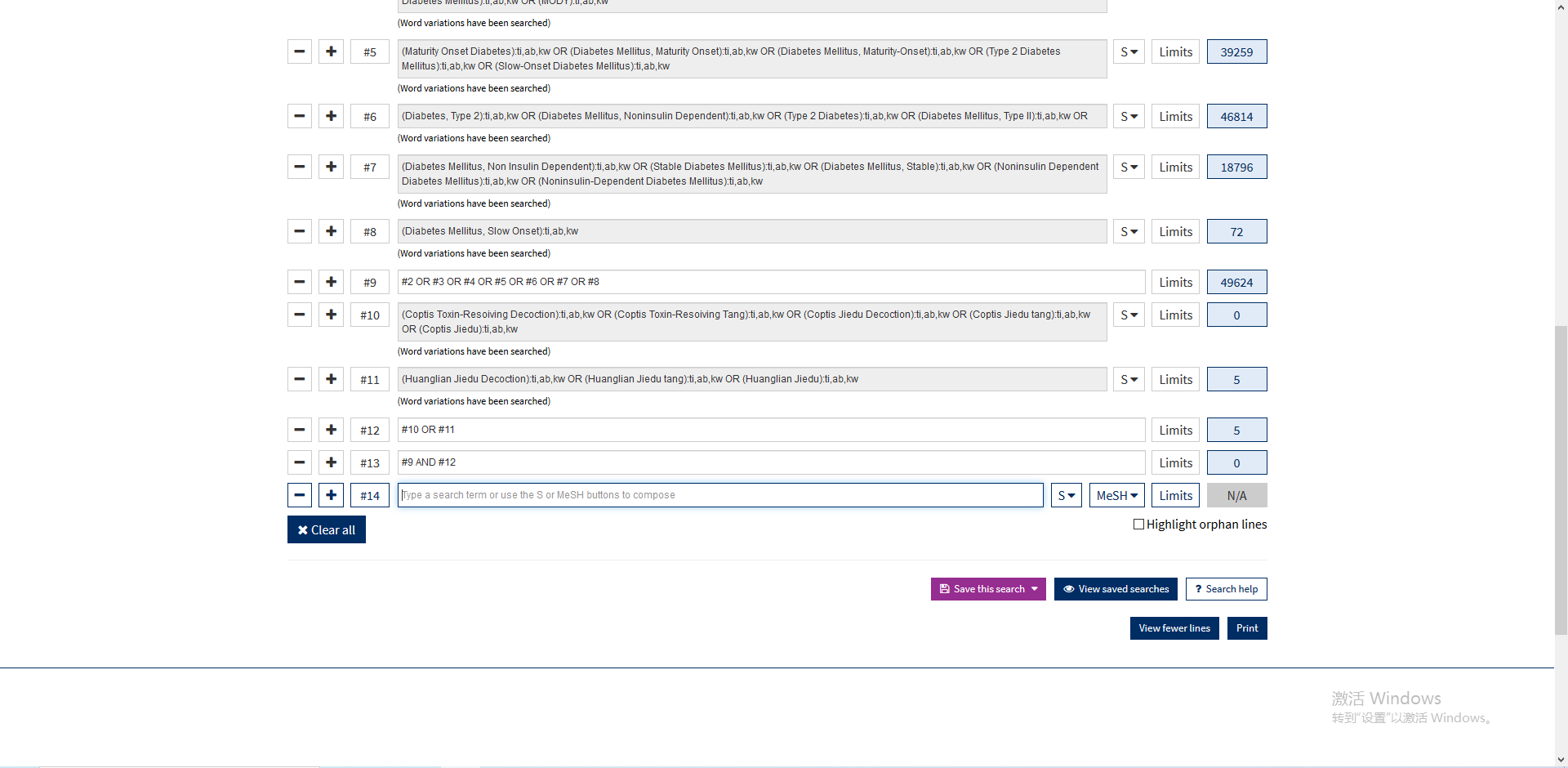
**

**CNKI**

**
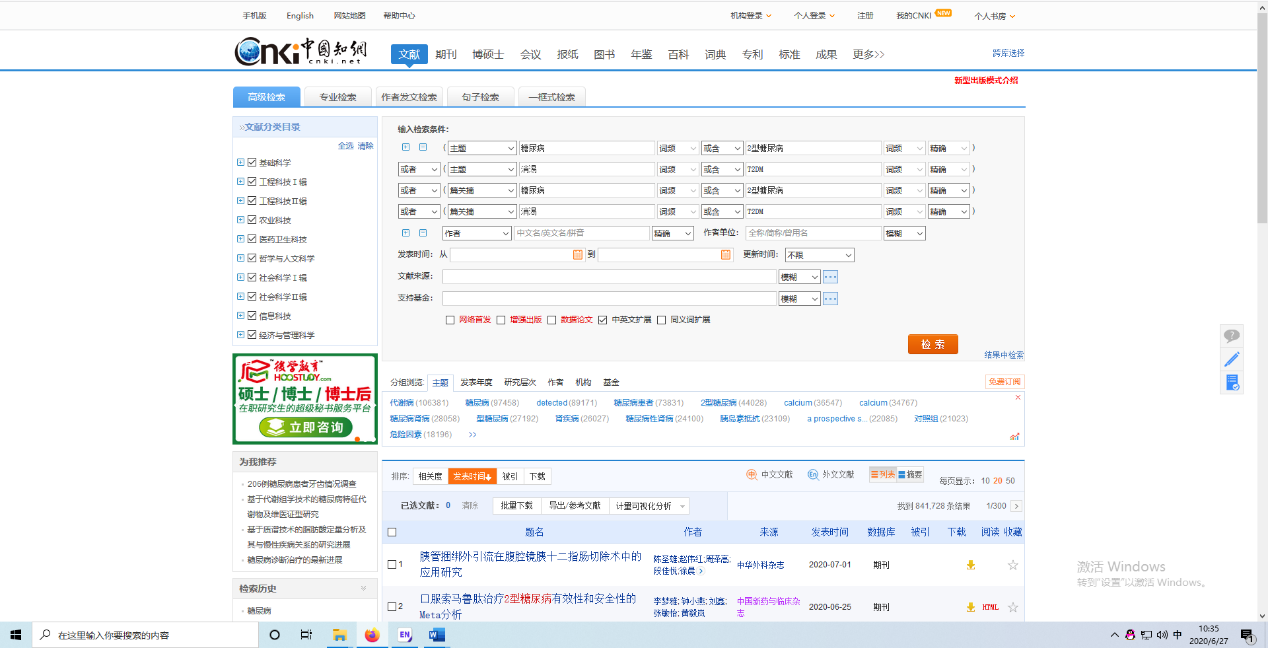

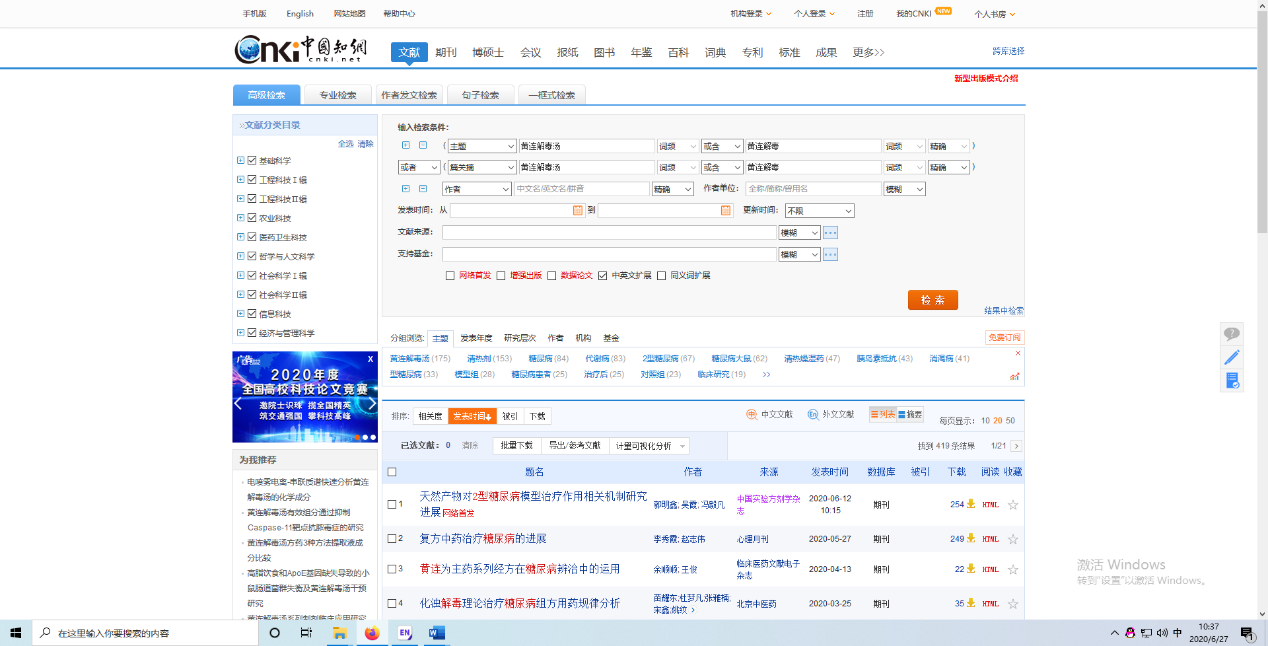
**

**Wanfang data**

**
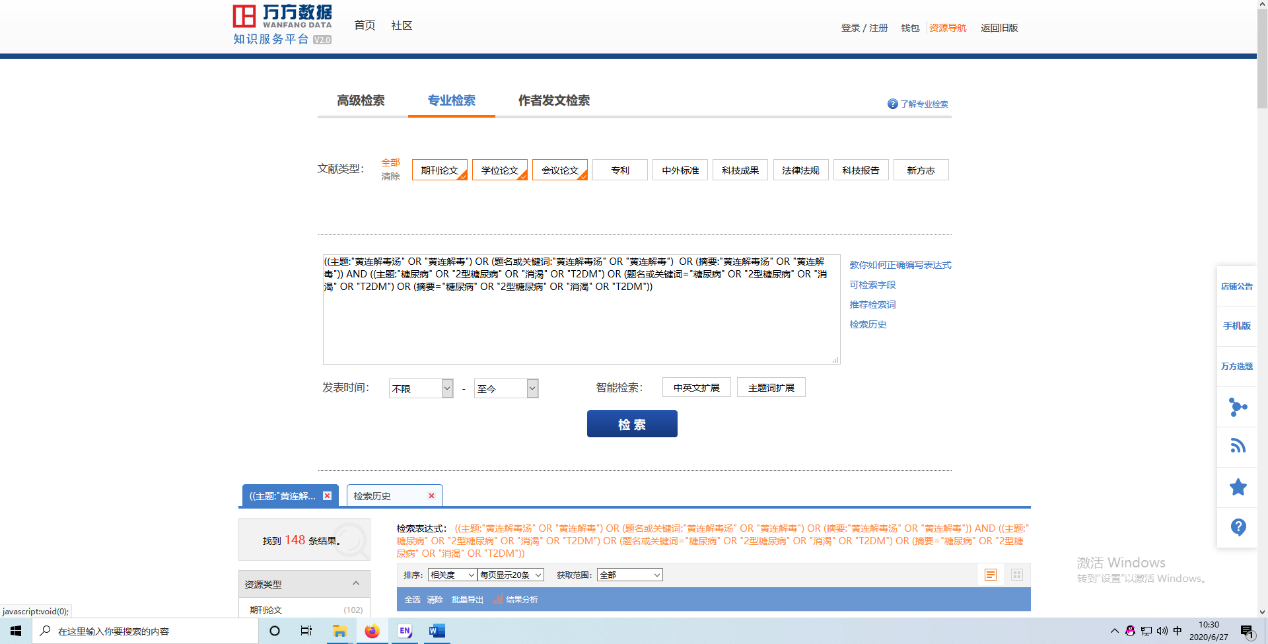
**

**Cqvip**

**
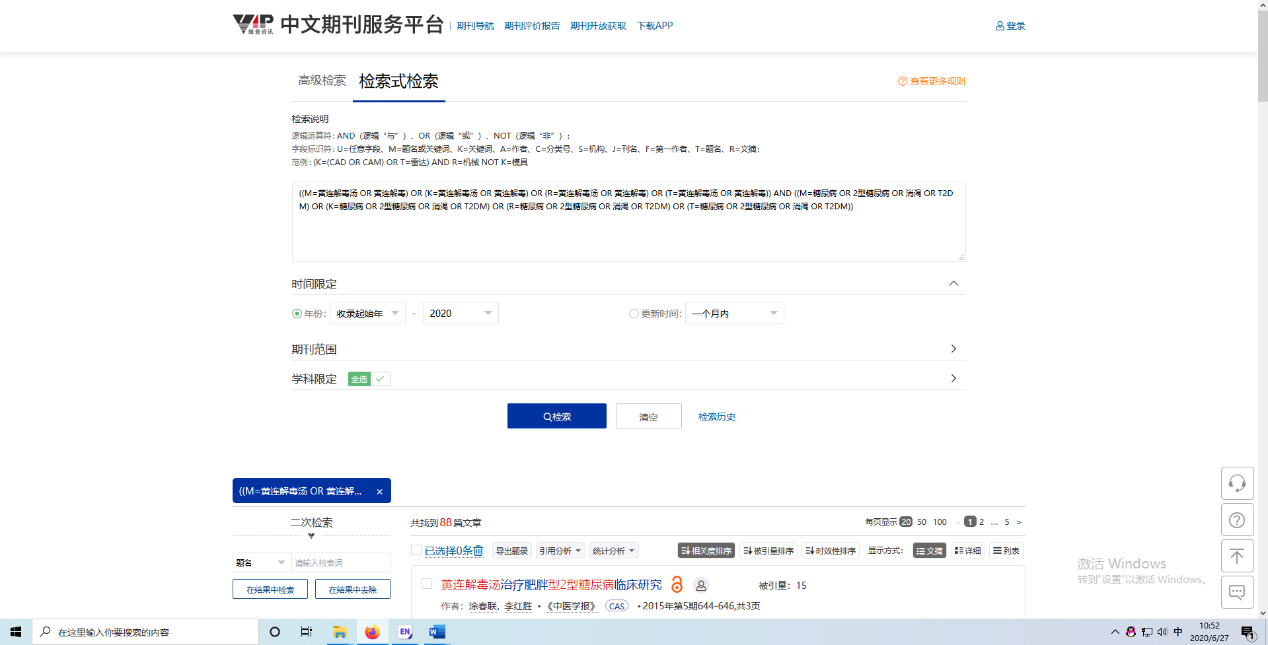
**

**ClinicalTrials.gov**

**
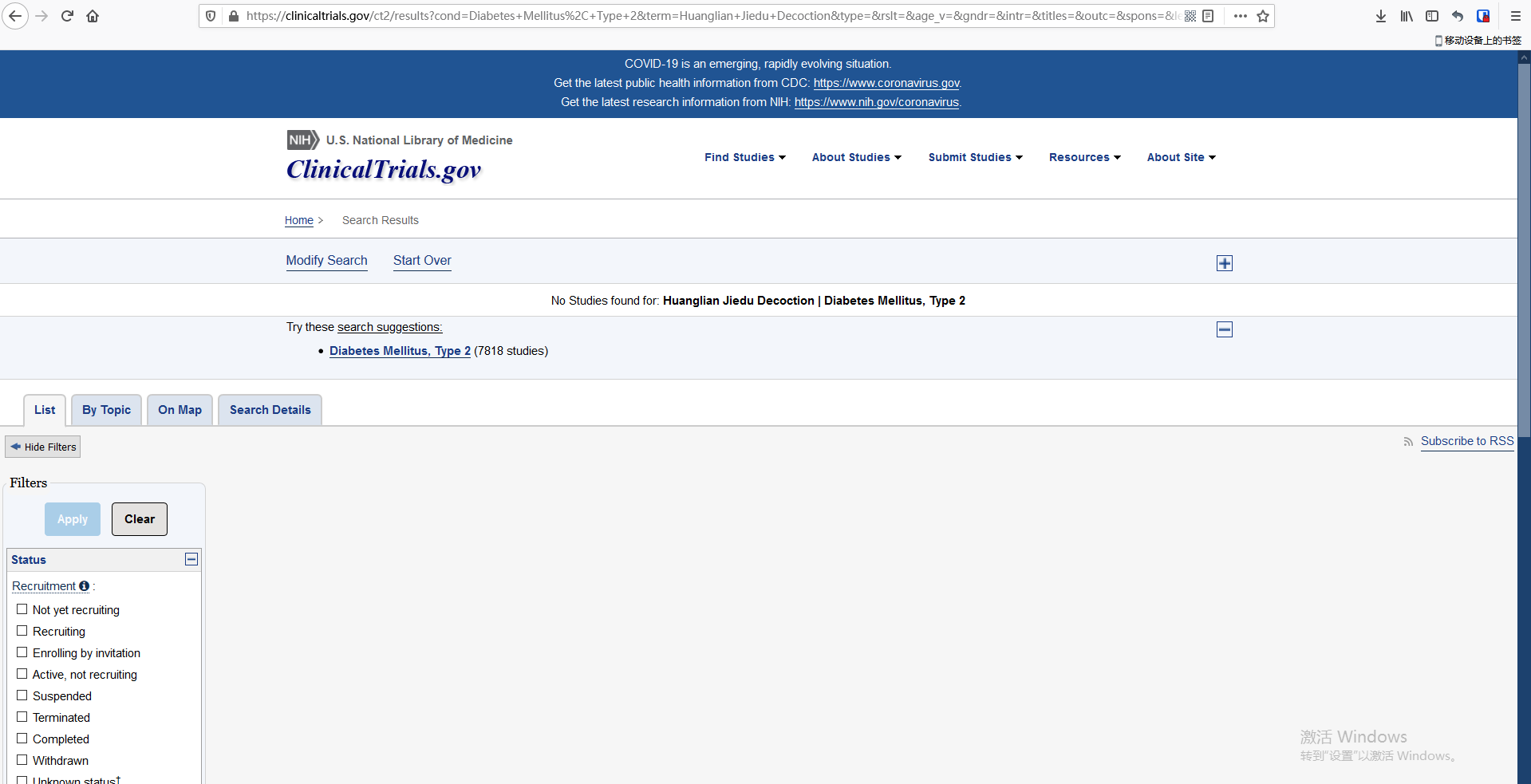
**

**ICTRP**

**
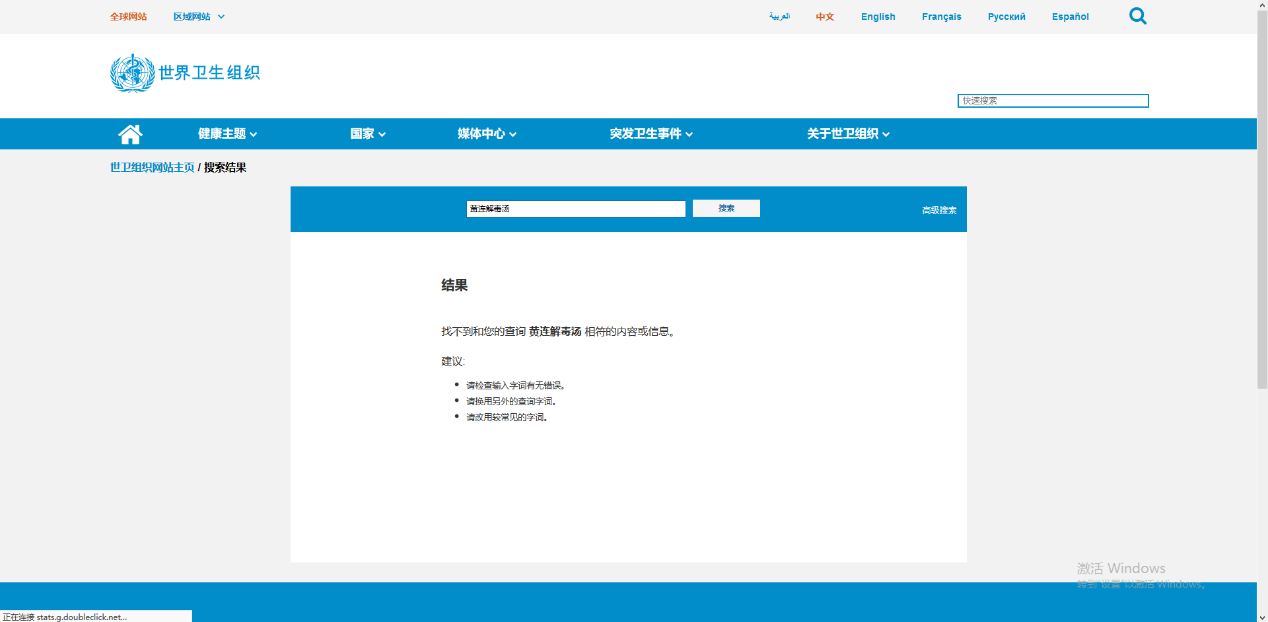
**

**CHiCTR**

**
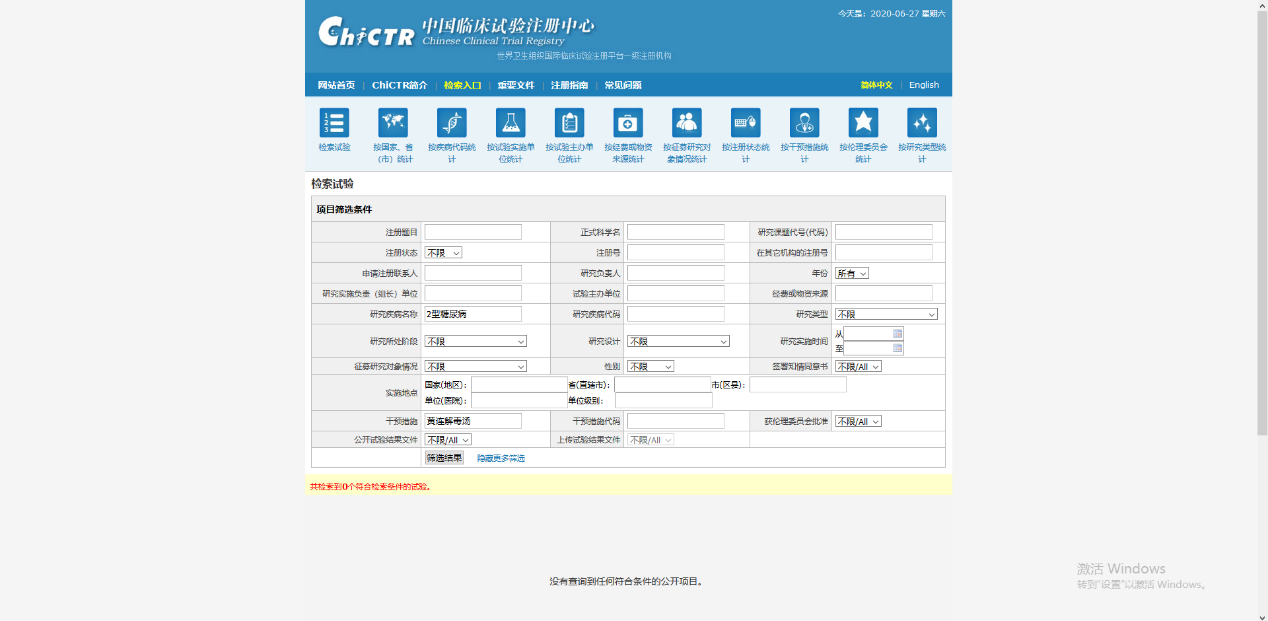
**
